# Computational Protein Design Quantifies Structural Constraints on Amino Acid Covariation

**DOI:** 10.1371/journal.pcbi.1003313

**Published:** 2013-11-14

**Authors:** Noah Ollikainen, Tanja Kortemme

**Affiliations:** 1 Graduate Program in Bioinformatics, University of California San Francisco, San Francisco, California, United States of America; 2 California Institute for Quantitative Biosciences (QB3), University of California San Francisco, San Francisco, California, United States of America; 3 Department of Bioengineering and Therapeutic Science, University of California San Francisco, San Francisco, California, United States of America; University of Texas at Austin, United States of America

## Abstract

Amino acid covariation, where the identities of amino acids at different sequence positions are correlated, is a hallmark of naturally occurring proteins. This covariation can arise from multiple factors, including selective pressures for maintaining protein structure, requirements imposed by a specific function, or from phylogenetic sampling bias. Here we employed flexible backbone computational protein design to quantify the extent to which protein structure has constrained amino acid covariation for 40 diverse protein domains. We find significant similarities between the amino acid covariation in alignments of natural protein sequences and sequences optimized for their structures by computational protein design methods. These results indicate that the structural constraints imposed by protein architecture play a dominant role in shaping amino acid covariation and that computational protein design methods can capture these effects. We also find that the similarity between natural and designed covariation is sensitive to the magnitude and mechanism of backbone flexibility used in computational protein design. Our results thus highlight the necessity of including backbone flexibility to correctly model precise details of correlated amino acid changes and give insights into the pressures underlying these correlations.

## Author Summary

Proteins generally fold into specific three-dimensional structures to perform their cellular functions, and the presence of misfolded proteins is often deleterious for cellular and organismal fitness. For these reasons, maintenance of protein structure is thought to be one of the major fitness pressures acting on proteins. Consequently, the sequences of today's naturally occurring proteins contain signatures reflecting the constraints imposed by protein structure. Here we test the ability of computational protein design methods to recapitulate and explain these signatures. We focus on the physical basis of evolutionary pressures that act on interactions between amino acids in folded proteins, which are critical in determining protein structure and function. Such pressures can be observed from the appearance of amino acid covariation, where the amino acids at certain positions in protein sequences are correlated with each other. We find similar patterns of amino acid covariation in natural sequences and sequences optimized for their structures using computational protein design, demonstrating the importance of structural constraints in protein molecular evolution and providing insights into the structural mechanisms leading to covariation. In addition, these results characterize the ability of computational methods to model the precise details of correlated amino acid changes, which is critical for engineering new proteins with useful functions beyond those seen in nature.

## Introduction

Evolutionary selective pressures on protein structure and function have shaped the sequences of today's naturally occurring proteins [1]–[3]. As a result of these pressures, sequences of natural proteins are close to optimal for their structures [4]. Natural protein sequences therefore provide an excellent test for computational protein design methods, where the goal is to predict protein sequences that are optimal for a desired protein structure and function [5]. It is often assumed that given a natural polypeptide backbone conformation, an accurate protein design algorithm should be able to predict sequences that are similar to the natural protein sequence. This test is commonly referred to as native sequence recovery [4] and it has been used extensively to evaluate various protein design sampling methods and energy functions [6]–[8].

Beyond simply recovering the native sequence, a further challenge in computational protein design is to predict the set of tolerated sequences that are compatible with a given protein fold and function [9]–[13]. Predicting sequence tolerance is important for applications such as characterizing mutational robustness [14], [15], predicting the specificity of molecular interactions [16]–[20], and designing libraries of proteins with altered functions [21], [22]. Recent methods developed for this goal involve generating an ensemble of backbone structures similar to the native structure and then designing low energy sequences for the different structures in the ensemble [9], [16], [19], [23]–[25]. These flexible backbone design methods can produce sequences that are highly divergent from the native sequence but may still fold into the desired structure, which makes simple native sequence recovery a poor indicator for the accuracy of these methods. A more useful computational test of these approaches involves comparing designed sequences with a set of reference sequences, either naturally occurring or experimentally derived, that share the desired protein fold. This comparison can be based on sequence profile similarity, which involves quantifying the difference between the frequencies of observing each amino acid at corresponding positions in the designed and reference sequences [16], [17], [19].

While high similarity between designed and reference sequence profiles can be informative to gauge the accuracy of a protein design method, it does not guarantee that the method will predict sequences that fold into the desired structure. This is because sequence profile comparisons evaluate amino acid positions independently from each other and therefore ignore the details of amino acid interactions that are critical for protein structure and function. Naturally occurring protein structures are formed cooperatively and each amino acid can physically interact with multiple neighboring amino acids. Evolutionary selective pressures have acted upon these interactions, resulting in the patterns of amino acid covariation that can be observed within today's naturally occurring protein families. Accordingly, previous studies have used information theoretic methods to detect amino acid covariation in multiple sequence alignments of many different protein families [26]–[28] and have used contact prediction based on covariation to dramatically improve the accuracy of protein structure modeling [29].

Despite the clear occurrence of amino acid covariation in natural protein sequences, the extent to which different selective pressures have shaped amino acid covariation in diverse protein families is unknown. Additionally, it is difficult to dissect to what extent phylogenetic bias has influenced the observations of amino acid covariation. Previous work has indicated that networks of covarying amino acids play a role in allosterically linking distant functional sites, suggesting that amino acid covariation is driven by protein functional constraints [30], [31]. However, other studies have shown in two test cases that computational protein design can recapitulate naturally occurring covariation in the cores of SH3 domains [4], [13], [32] and for two-component signaling systems [33]. These results indicate that constraints imposed by protein structure have played a role in producing the covariation in the studied examples, but it has not yet been shown that these observations are general.

In this paper, we use computational protein design to measure the extent to which protein structure has shaped amino acid covariation in a diverse set of 40 protein domains. Since computational protein design predicts sequences that are energetically optimal based on protein structure alone, we expect that pairs of amino acids that highly covary in both designed and natural sequences to have likely covaried to maintain protein structure. We find significant overlap in the sets of highly covarying amino acid pairs between designed and natural sequences for all 40 domains examined, suggesting that maintenance of protein structure is a dominant selective pressure that constrains the evolution of amino acid interactions in proteins. Our analysis furthermore quantifies the extent to which different types of interactions explain the observed covariation. Finally, we demonstrate the utility of amino acid covariation recapitulation as a sensitive test for evaluating different protein design methods. We find that flexible backbone design significantly improves covariation recapitulation relative to fixed backbone design and that recapitulation of amino acid covariation is exquisitely sensitive to both the magnitude and mechanism of backbone flexibility. Taken together, these results provide fundamental insights into the physical nature of amino acid co-evolution and, more practically, provide a new benchmark that may help improve the accuracy of computational protein design methods.

## Results

### Computational protein design recapitulates natural amino acid covariation

To compare amino acid covariation in natural and predicted designed protein sequences, we selected 40 protein domains that were diverse with respect to their secondary structure composition and fold class (Table 1). We then quantified natural amino acid covariation for each domain by creating a multiple sequence alignment for the domain, followed by computing covariation between every pair of columns in the multiple sequence alignment by using a mutual information based method [28] (see Methods). Pairs of amino acid positions with a covariation score that is two standard deviations above the mean or greater were considered to be highly covarying pairs.

**Table 1:** Protein domains used in this study.

Pfam ID	Pfam Name	Total # of sequences	PDB ID	Amino acid length	SCOP class
PF00226	DnaJ	19122	2O37	60	a
PF00249	Myb_DNA-binding	9398	1GUU	47	a
PF00439	Bromodomain	4483	3JVL	89	a
PF00486	Trans_reg_C	35180	2ZXJ	77	a
PF00550	PP-binding	28748	1T8K	68	a
PF01029	NusB	5275	1TZV	127	a
PF01035	DNA_binding_1	5230	3GVA	87	a
PF01627	Hpt	7684	2A0B	88	a
PF12844	HTH_19	9622	3FYM	70	a
PF00072	Response_reg	103232	1MVO	111	a/b
PF00085	Thioredoxin	16281	1FB0	104	a/b
PF00581	Rhodanese	19885	1GN0	92	a/b
PF00582	Usp	15546	2Z3V	137	a/b
PF01451	LMWPc	5201	1JL3	130	a/b
PF00013	KH_1	11484	1WVN	63	a+b
PF00076	RRM_1	31837	2X1B	71	a+b
PF00111	Fer2	11941	1CZP	76	a+b
PF00179	UQ_con	6107	1Z2U	138	a+b
PF00240	Ubiquitin	8316	2BWF	69	a+b
PF00254	FKBP_C	11034	2PPN	95	a+b
PF00327	Ribosomal_L30	3324	1BXY	52	a+b
PF00381	PTS-HPr	5246	1PTF	84	a+b
PF00542	Ribosomal_L12	3474	1CTF	68	a+b
PF00691	OmpA	11815	1OAP	96	a+b
PF00708	Acylphosphatase	3057	3BR8	89	a+b
PF04002	RadC	3253	2QLC	123	a+b
PF00018	SH3_1	8993	2O9S	48	b
PF00041	Fn3	26172	1TEN	81	b
PF00168	C2	11697	3F04	87	b
PF00169	PH	8137	1UNQ	103	b
PF00313	CSD	9848	3I2Z	67	b
PF00355	Rieske	9153	1FQT	95	b
PF00364	Biotin_lipoyl	16853	2EVB	68	b
PF00498	FHA	7247	3GQS	67	b
PF00595	PDZ	12568	2H3L	87	b
PF01833	TIG	4814	3MQI	83	b
PF02823	ATP-synt_DE_N	3782	1AQT	80	b
PF07679	I-set	29272	1U2H	91	b
PF07686	V-set	9255	2PND	116	b
PF08666	SAF	3256	1UCS	60	b

Forty diverse protein domains were selected from Pfam. This table contains the Pfam information for each domain, the total number of sequences assigned to this domain according to Pfam, the PDB ID of the domain crystal structure used for design, the domain length and the SCOP classification.

We predicted designed protein sequences for each of the 40 domains using RosettaDesign [4], [34]. We first used the standard RosettaDesign fixed backbone protocol [34], which takes a crystal structure as input and runs Monte Carlo simulated annealing, to predict 500 designed sequences for each domain structure. We then quantified amino acid covariation in the designed sequences and compared it to natural amino acid covariation for each domain. We calculated the similarity between designed and natural covariation based on the percent overlap of the highly covarying pairs in each set (see Methods). We found this overlap to be significant (p<0.001) for all 40 domains (Table S1).

### Magnitude of structural variation affects covariation similarity

**Figure 1:**
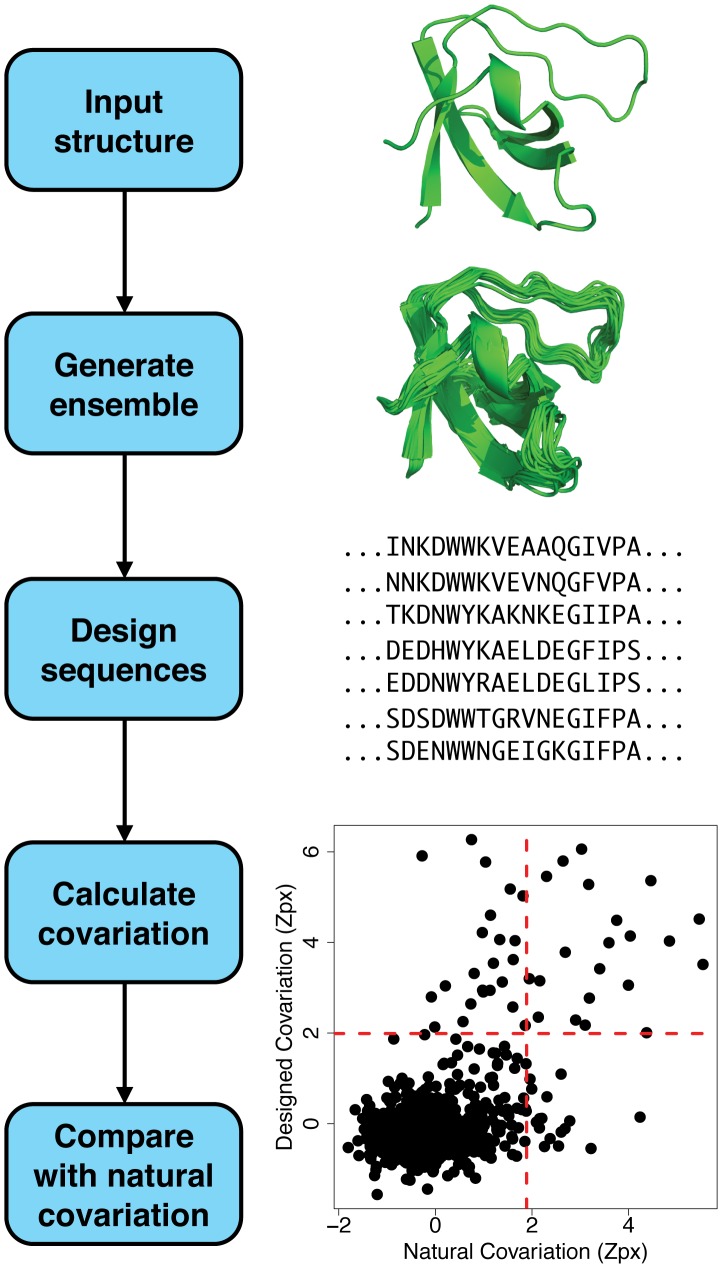
Flow chart of the computational strategy to compare natural and designed amino acid covariation. For each domain family (the SH3 domain in the example), a crystal structure of the domain is obtained from the Protein Data Bank. This structure is used as input to a protocol that generates a conformational ensemble of protein structures. Each structure in this ensemble is then input to a protocol that designs a low energy sequence consistent with the structure. Amino acid covariation is calculated for every pair of positions in the designed sequences, and the designed covariation is compared to the covariation seen among naturally occurring sequences with the same protein domain.

Given the observation that fixed backbone protein design can recapitulate a significant fraction of naturally covarying amino acid pairs, we next aimed to understand how incorporating backbone flexibility into the design protocol affects this recapitulation. To accomplish this, we generated a conformational ensemble of 500 backbone structures for each domain using the “backrub” method [35] in Rosetta [36], which iteratively applies local backbone perturbations throughout the protein structure combined with adjustments in side-chain conformations. We then used RosettaDesign to predict a low energy sequence for each backbone structure in the ensemble, resulting in 500 designed sequences. Figure 1 shows a flow chart of this approach applied to an SH3 domain.

**Figure 2:**
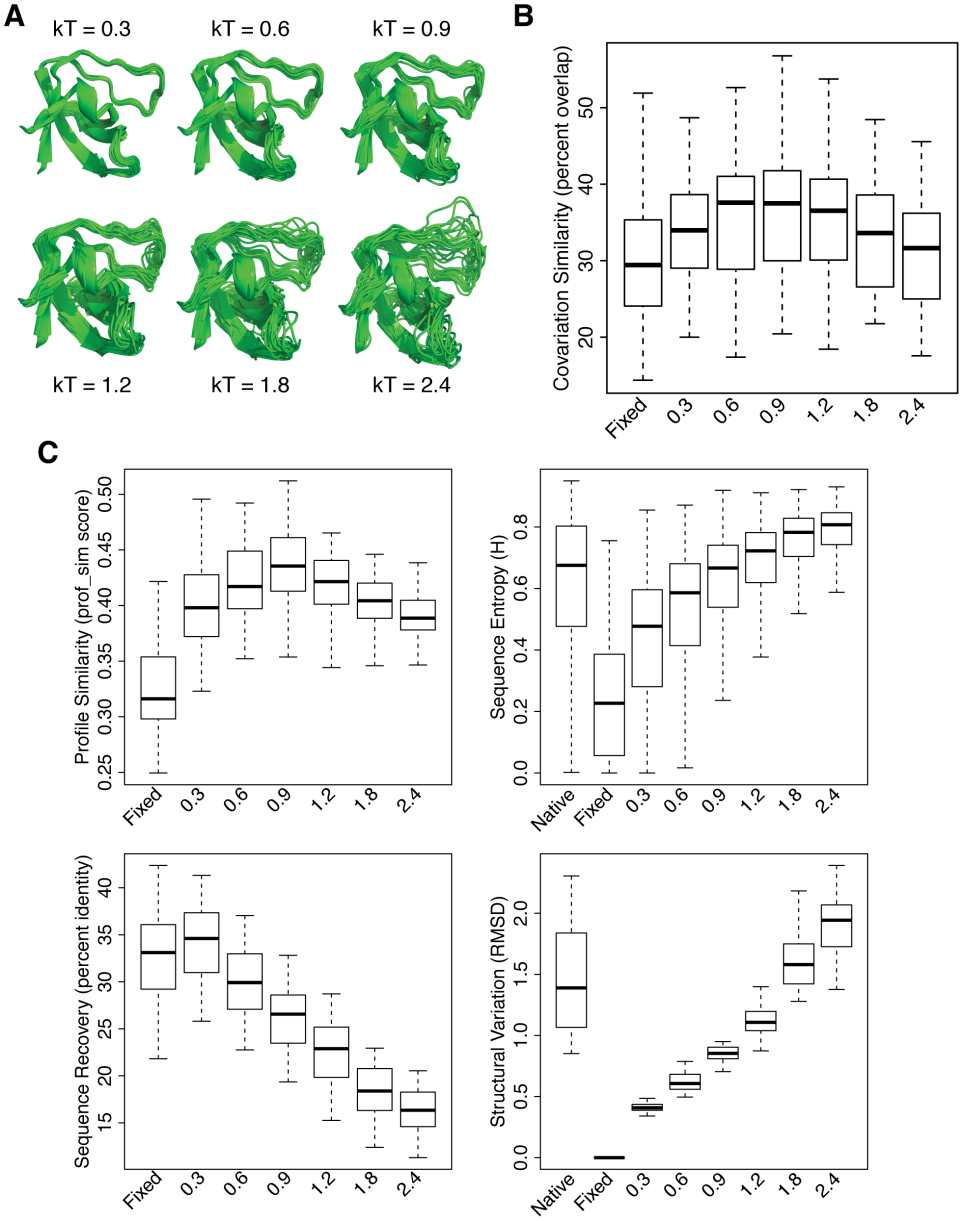
Effects of the magnitude of structural variation on sequence properties. A) Representative structures of conformational ensembles generated using backrub Monte Carlo simulations with different temperature parameters (shown for an SH3 domain). B) Box plot showing the distributions of covariation similarity values between natural sequences and sequences designed using conformational ensembles generated with different temperature parameters (including “Fixed” backbone design sequences). Each distribution contains 40 covariation similarity values, one for each of the 40 protein domains. For each box plot in this study, the top and bottom whiskers denote the maximum and minimum values, respectively. The top and bottom of the box indicates the 75th and 25th percentiles, respectively, and the bold line denotes the 50th percentile. C) Box plots showing the distributions of sequence and structural characteristics for each temperature. The sequence profile similarity, sequence recovery and structural variation distributions each contain 40 values, one for each of the 40 protein domains. The sequence entropy distributions each contain 2778 values, one for each position in the 40 protein domains. “Native” distributions for structural variation and sequence entropy of the natural proteins were included as well.

To investigate the effect of the magnitude of backbone flexibility in the design protocol, we varied the temperature parameter in the Monte Carlo simulations used in the backrub protocol to generate conformational ensembles with different amounts of structural variation (Figure 2A). We designed sequences for each ensemble (kT = 0.3, 0.6, 0.9, 1.2, 1.8, 2.4) and quantified similarity to natural covariation for each set of sequences. We compared these results with sequences designed using the fixed backbone design protocol described above (“Fixed”). Figure 2B shows a significant increase in covariation similarity for the flexible backbone simulations relative to the fixed backbone simulation. Moreover, the distributions of covariation similarity for the 40 domains show that there is an optimal degree of structural variation, as low-temperature and high-temperature simulations perform significantly worse than mid-temperature simulations (Table S2). We observed this same trend when we repeated this analysis using a different method for quantifying covariation [37] (Figure S1), suggesting that our results are not dependent on the method used to quantify covariation.

To better understand the basis of this trend, we examined several other sequence and structural characteristics: sequence recovery, sequence profile similarity, sequence entropy and structural variation (see Methods). The resulting distributions for these characteristics are shown in Figure 2C. Sequence entropy and sequence profile similarity showed similar trends to covariation similarity (sequence entropy is most similar to natural sequences and profile similarity is highest at 0.9 kT), suggesting that backbone flexibility allows for sampling diverse sequences with native-like properties. These trends are consistent with the observation that sequence recovery decreases with increasing amounts of backbone flexibility. As diversity within a set of sequences increases, those sequences tend to become more dissimilar to any individual sequence, including the native sequence of the crystal structure used as input for design. Structural variation in the 0.3, 0.6, 0.9 and 1.2 kT simulations is less than the structural variation among naturally occurring protein structures with these domains, which could be due to the fact that natural proteins use additional mechanisms of generating structural variation that are not being modeled, such as the insertion or deletion of amino acids in loop regions. Taken together, these results suggest that a moderate degree of backbone flexibility allows for the accommodation of sequences that differ from the native sequence and yet are similar to naturally occurring sequences with respect to their sequence profiles, sequence entropies and patterns of amino acid covariation.

### Mechanism of structural variation affects covariation similarity

Next we examined whether or not these results were specific to the method used to generate the conformational ensembles for design. We tested two other Monte Carlo based methods that iteratively perform perturbations to the backbone. One method performed Kinematic Closure (“KIC”), which involves randomizing phi/psi torsions in a local region of the backbone while keeping the rest of the backbone fixed, thus introducing a chain break, and then using inverse kinematics to solve for the torsions that will close the chain [38]. The other method performs potentially non-local moves by perturbing the phi and psi torsions of residues by a random small angle (“Small”) [39]. We ran both of these methods for the same number of trials and for the same values of kT as the backrub protocol. The resulting distributions of covariation similarity show the same trend we observed previously with the backrub simulations, where mid-range temperature simulations result in an optimal degree of covariation similarity (Figure S2).

**Figure 3:**
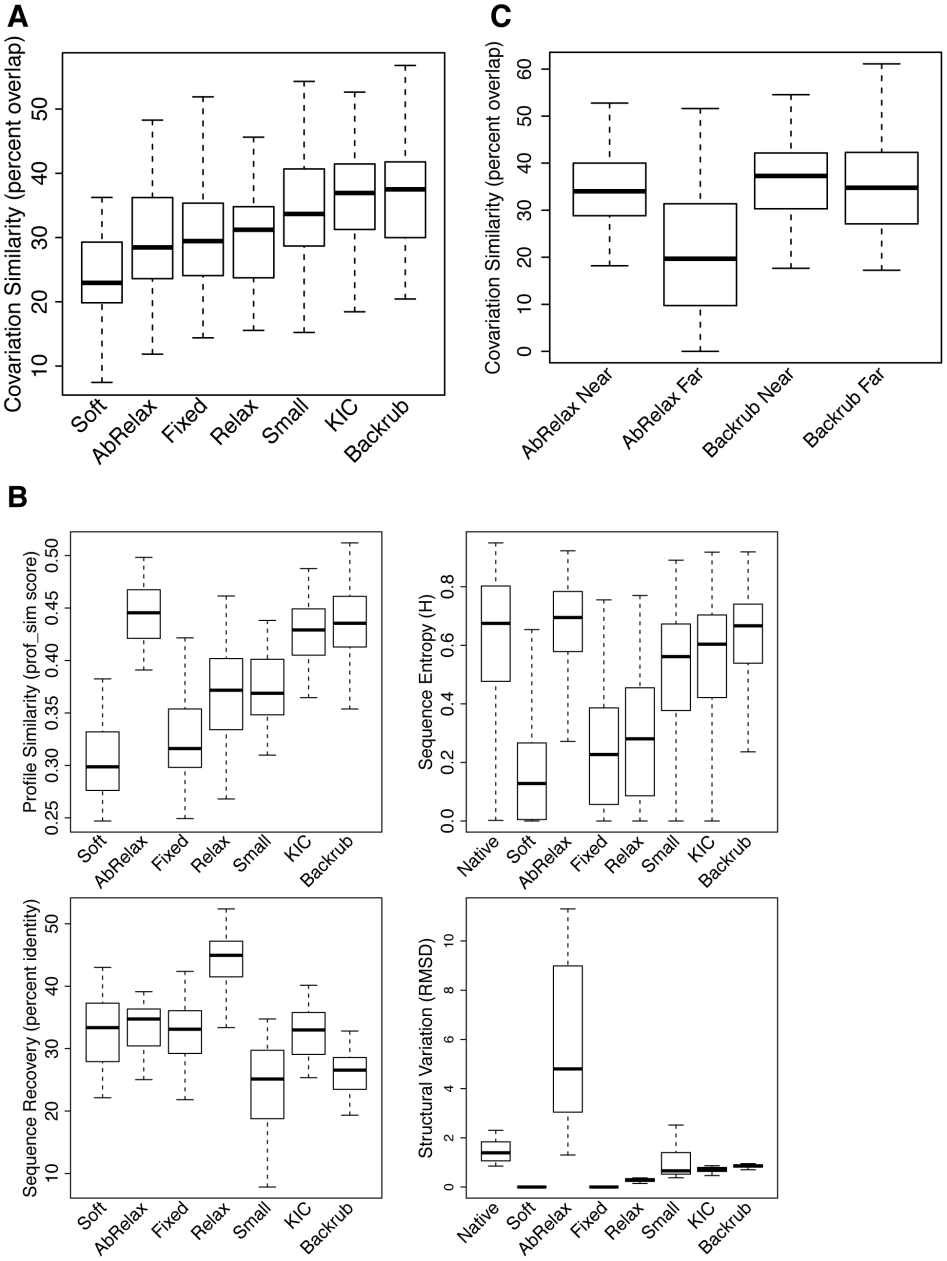
Effects of the mechanism of structural variation on sequence properties. A) Box plot showing the distributions of covariation similarity values between natural sequences and sequences designed using conformational ensembles generated with different methods. The Backrub, KIC and Small simulations shown here were run with kT values of 0.9, 1.2, and 1.2, respectively (which represents the optimal temperature for covariation similarity in each case, see Figures 2B and S2). B) Box plots showing the distributions of sequence and structural characteristics for each method of generating conformational ensembles. C) Covariation similarity distributions for subsets of covarying pairs that are “near” in sequence (separated by 10 residues or fewer) or “far” in sequence (separated by greater than 10 residues).

While the optimal simulation temperature parameter was comparable for each of the methods tested, the methods achieved a different optimum level of covariation similarity with the natural sequences. We found that the two local move simulations (KIC and Backrub) outperformed the non-local move simulation (Small). To test if this observation holds true more generally, we tested two additional methods of generating conformational ensembles that make non-local moves. These methods included FastRelax (“Relax”), which consists of multiple rounds of side-chain repacking and all-atom minimization while increasing the weight of the repulsive term in the Lennard–Jones (LJ) potential from 2% to 100% of its default value, and AbInitioRelax (“AbRelax”), which performs fragment-based *ab initio* structure prediction followed by FastRelax [40]. As an additional control, we also designed sequences using a fixed backbone structure with an energy function that dampens the weight of the repulsive LJ term (“Soft”). The resulting covariation similarity distributions show that recapitulation of natural amino acid covariation is sensitive to the method used to generate conformational ensembles (Figure 3A). Both local move simulations (KIC, Backrub) achieved higher median covariation similarities than the non-local move simulations (Small, AbRelax, Relax) and the fixed backbone simulations (Fixed, Soft) (see Table S3 for p-values).

We also evaluated each of these methods using the other metrics described above: native sequence recovery, sequence profile similarity, sequence entropy and structural variation (Figure 3B). Unexpectedly, the AbRelax method, which resulted in conformational ensembles with the greatest structural variation, achieved the highest sequence profile similarity with the natural sequences of any method tested. A possible explanation for this behavior is that local interactions are preserved in AbRelax generated structures, but the overall topology of the protein is incorrect. To test this hypothesis, we examined covariation similarity in the AbRelax sequences by splitting all covarying pairs into the following two sets: pairs separated by fewer than 10 residues in sequence (“Near”) and pairs separated by greater than 10 residues in sequence (“Far”). This analysis revealed that whereas AbRelax sequences have relatively high covariation similarity with natural sequences for pairs close in sequence, they have low covariation similarity for pairs that are distant in sequence (Figure 3C). In contrast, covariation similarity for “near” and “far” pairs were similar for simulations using backrub ensembles. These results suggest that AbRelax can model local interactions within a secondary structural element or between adjacent secondary structures, but it does not correctly capture non-local interactions that are likely critical for achieving a cooperatively folded, stable tertiary structure. This observation demonstrates the importance of using amino acid covariation to evaluate the accuracy of protein design methods, since it is possible to obtain deceptively high sequence profile similarity scores with highly divergent tertiary structures as long as local interactions are maintained. Of all the flexible backbone design methods tested, Backrub, kT = 0.9 resulted in sequences most similar to the natural sequences with respect to covariation similarity and sequence profile similarity. Using the assumption that a method that gives higher similarity to natural sequences will better capture the mechanisms underlying covariation, we used Backrub, kT = 0.9 as the representative flexible backbone sequences for the remainder of the study.

### Backbone flexibility allows for amino acid interactions that fixed backbones cannot accommodate

**Figure 4:**
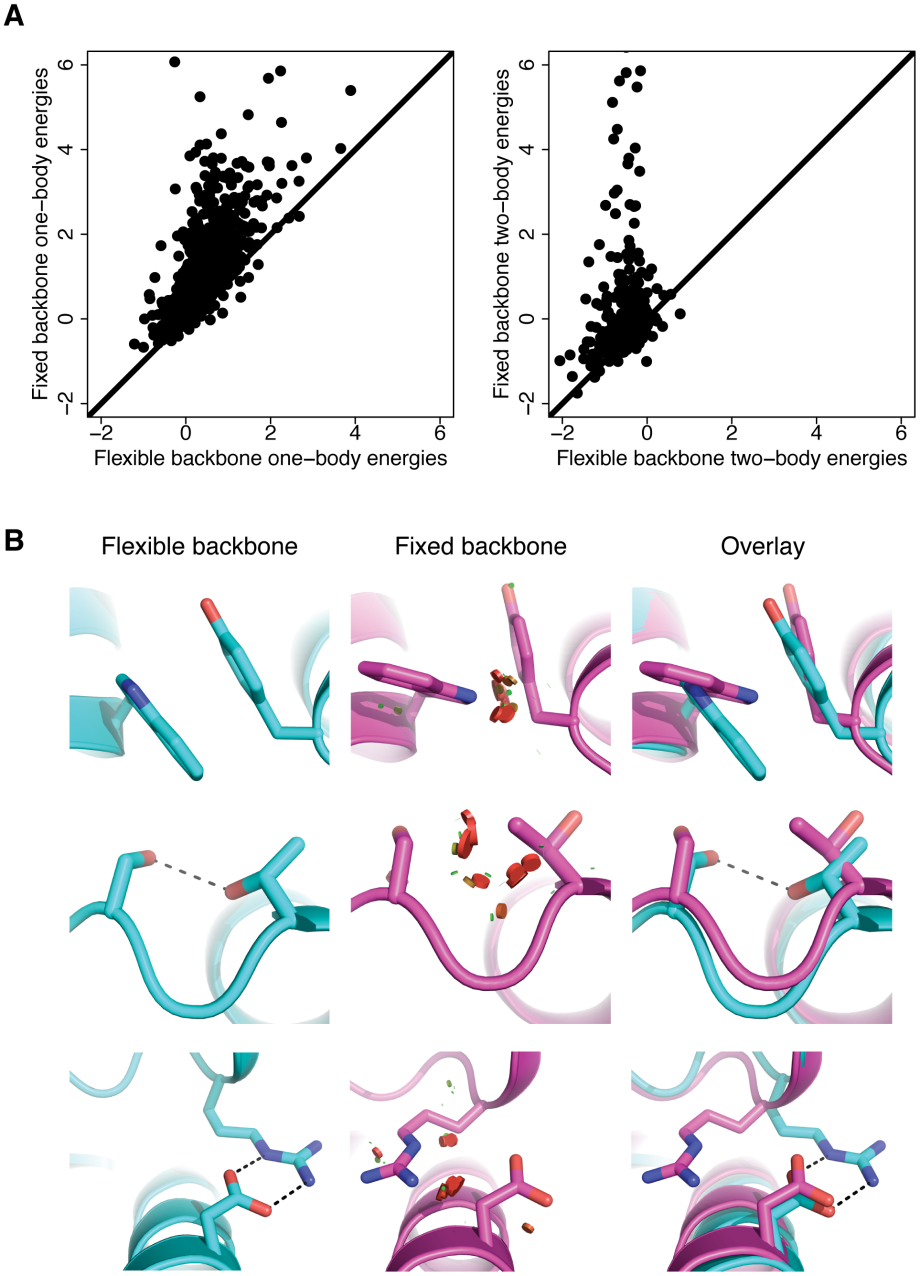
Energetic effects of forcing amino acid covariation onto fixed backbones. A) Scatter plots of covarying pair energies in the context of fixed or flexible backbones. Each dot represents a pair of positions that was found to be highly covarying in the flexible backbone sequences (Backrub, kT = 0.9) and the natural sequences but not in the fixed backbone sequences. Pairs of amino acids at these positions that were found in flexible backbone designs but not in fixed backbone designs were forced onto fixed backbones taken from X-ray crystal structures and their one and two-body energies were calculated. The left plot shows a comparison of one-body energies and the right plot shows a comparison of two-body energies for these pairs. B) Representative examples of pairs of amino acids that require backbone movements to achieve low-energy interactions. Models from flexible backbone design (Backrub, kT = 0.9) are shown in cyan and models from fixed backbone design are shown in magenta. The top case shows a ring stacking interaction, the middle case shows a hydrogen bonding interaction and the bottom case shows a salt bridge interaction. Red disks represent steric clashes, where the radius and number of the disks is proportional to the magnitude of the clash.

To understand how backbone flexibility influences the extent of covariation similarity between designed and natural sequences, we identified all pairs of amino acid positions that highly covaried in both the natural sequences and a set of flexible backbone sequences (Backrub, kT = 0.9) but did not highly covary in the fixed backbone sequences. We then took all pairs of amino acids at these positions that were not sampled in the fixed backbone simulation and designed them onto the crystal structure backbone using fixed backbone design. For each pair of these positions, we calculated mean interaction energies and compared these energies between fixed and flexible backbone design structures (Figure 4A). We calculated both one-body energies, which include the interaction of an amino acid residue with itself, and two-body energies, which include the interactions between two amino acid residues in the protein (see Text S1 for description of the components of Rosetta one-body and two-body energies). We found both the one-body and two-body energies of these pairs to be generally greater in the context of fixed backbones relative to flexible backbones. Splitting the energies into their component terms revealed that the backbone-dependent Dunbrack rotamer energy (fa_dun) and Lennard-Jones repulsive (fa_rep) terms resulted in greater energy increases in the one-body and two-body energies, respectively, than any other term in the energy function (Figure S3). These results suggest that amino acid pairs that covary in flexible backbone simulations but do not covary in fixed backbone simulations generally cannot be accommodated on fixed backbones without resulting in steric clashes or rotamers that are unfavorable for the given backbone. Simply modifying the energy function by using a “soft” repulsive potential that reduces the energy of clashes does not increase sequence diversity or covariation similarity (Figure 3B), suggesting that backbone movements are required to accommodate these amino acid interactions. Figure 4B shows representative cases where some degree of backbone flexibility is required to correctly model the precise interaction details of specific amino acid pairings.

### Natural and designed amino acid pair propensities are highly correlated

**Figure 5:**
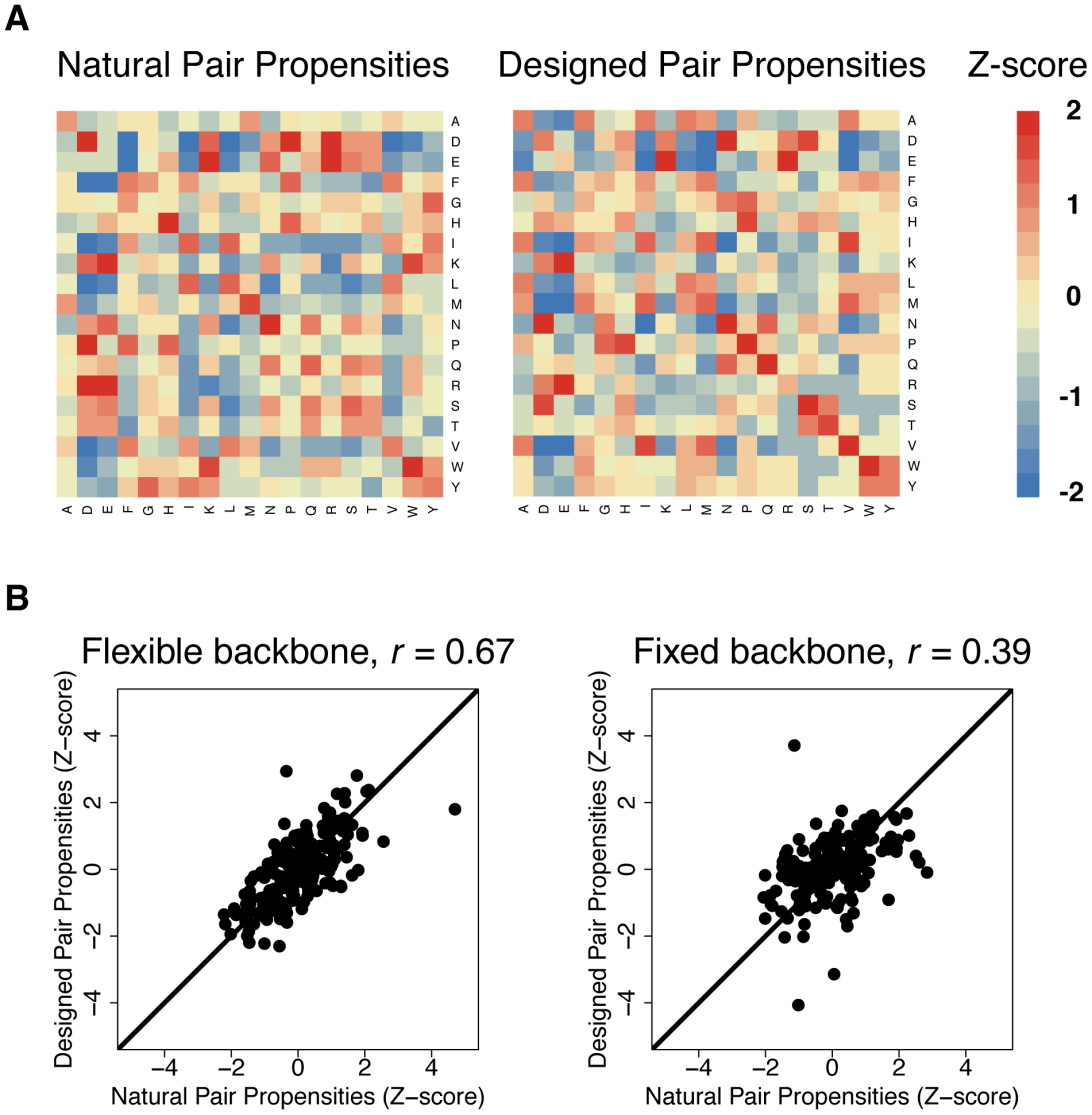
Correlation of amino acid pair propensities between natural and designed covarying pairs. A) Heat maps of amino acid pair propensities at pairs of positions that are highly covarying in both designed and natural sequences. Red pairs are over-represented at covarying positions and blue pairs are under-represented at covarying positions. The values are shown as Z-scores, which denote the number of standard deviations above or below the mean. B) Correlation of amino acid pair propensity Z-scores between designed and natural sequences. The left plot shows the correlation from flexible backbone design sequences and the right plot shows the correlation from fixed backbone design sequences. A Pearson correlation coefficient (r) is shown for each plot.

We have thus far compared amino acid covariation between natural and predicted designed sequences based on the extent of overlap between the sets of highly covarying pairs. However, it is also important to consider the amino acid pair propensities at covarying positions to test whether the natural and designed covarying pairs utilize the same types of amino acid interactions. To accomplish this, we calculated amino acid propensities at pairs of positions that covary in both the natural and designed sequences (Figure 5A). Over-represented amino acid pairs in both designed and natural sequences included those with opposite charges, hydrophobic pairs and hydrogen-bonding pairs. Differences in the designed and natural amino acid pair propensities included the over-representation of cation-pi pairs in the natural sequences but not in the designed sequences (such as W-R). These differences highlight shortcomings of the energy function used for design, which does not currently account for cation-pi interactions.

To quantify the similarity between the natural and designed covarying pair propensities, we calculated the correlation coefficients between the natural and designed propensities for all sets of designed sequences. We found these correlations to be dependent on both the magnitude and mechanism of backbone flexibility, as we previously observed with the overlap in covarying pairs (Table S4). The comparison between natural and designed pair propensities for fixed backbone sequences and for a set of flexible backbone sequences (Backrub, kT = 0.9) are shown in Figure 5B, again supporting the conclusion that backbone flexibility improves recapitulation of amino acid covariation.

### Mechanisms underlying covariation in natural and design sequences include complementary changes in amino acid size, charge or hydrogen bonding

**Figure 6:**
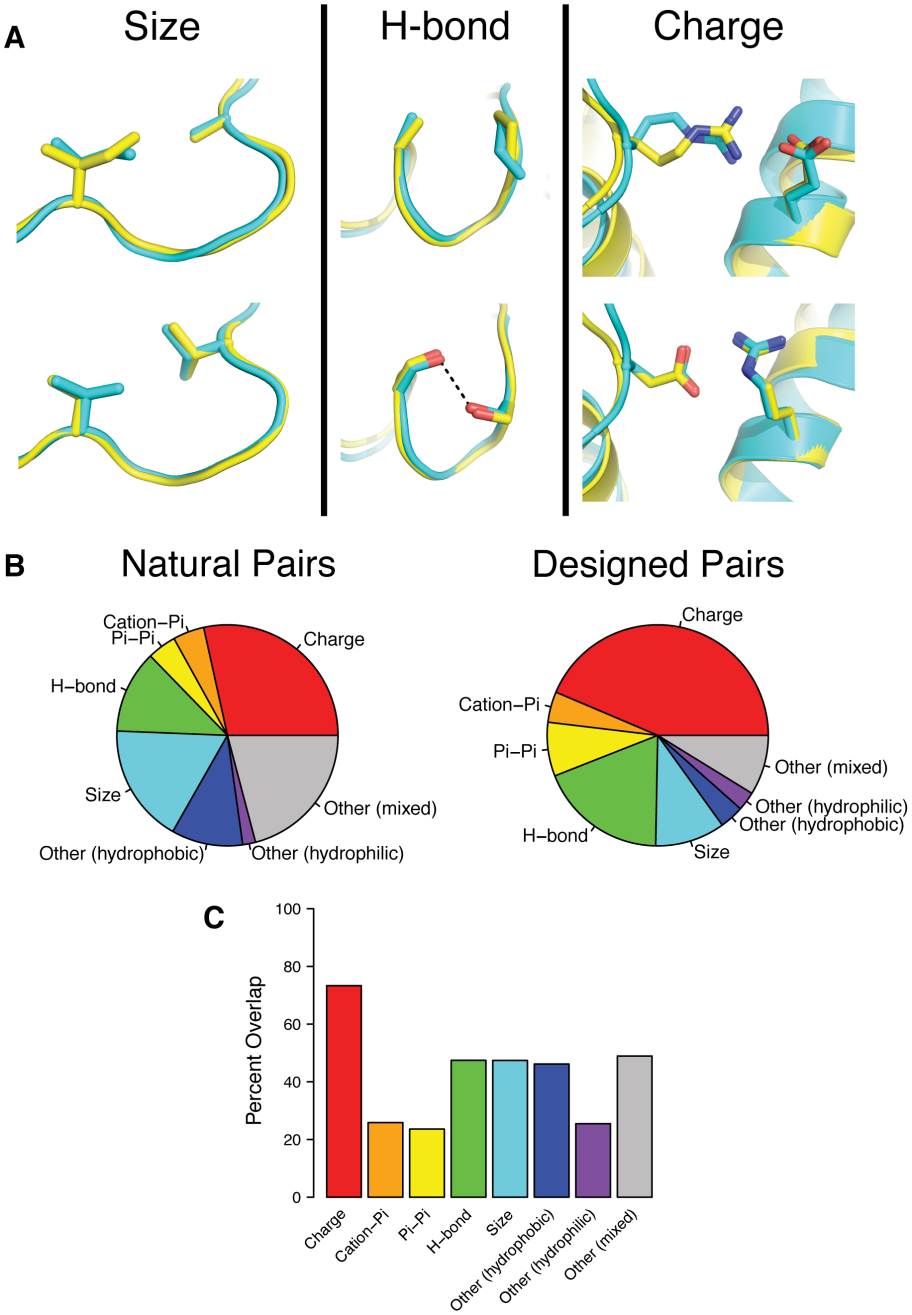
Covariation mechanisms of natural and designed covarying pairs. A) Representative examples of covariation mechanisms in both natural and designed sequences. Models from flexible backbone design (Backrub, kT = 0.9) are shown in cyan and x-ray crystal structures are shown in yellow. The left panel shows covariation due to size, the middle panel shows covariation due to hydrogen bonding and the right case shows covariation due to charge. The top and bottom rows for each panel show different amino acid pairs in the same positions but in different proteins. B) Pie charts showing the distribution of covariation mechanisms for pairs that covary in both natural and designed sequences. The left pie chart shows covariation mechanisms for natural pairs and the right shows covariation mechanisms for designed pairs. See the methods for a definition of the mechanism classification. C) Bar plot showing the percent overlap between natural and designed pairs for each covariation mechanism.

While similar pair propensities between natural and designed covarying pairs demonstrate that the same types of amino acid interactions occur in both natural and designed sequences, they do not show that the mechanisms underlying covariation are the same in both cases. To investigate this, we first classified the mechanism of covariation for all pairs that covary in both designed and natural sequences and then quantified how often the same mechanism is used. Figure 6A shows an illustration of three of the covariation mechanisms: size, hydrogen bonding and charge. Classifying each of these mechanisms requires examining the transition from one amino acid pair to another. For example, the transitions depicted in Figure 6A are IA–VV, AP–SS, RE–DR. Covariation due to size involves a decrease in the size of one amino acid and an increase in the size of the other (IA–VV). Covariation due to hydrogen bonding involves a hydrogen bond that exists in one pair but not the other (AP–SS). Covariation due to charge involves a pair of amino acids with opposite charges that either swap sign (RE–DR) or become uncharged amino acids. We also defined covariation mechanisms based on cation-pi interactions, pi-pi interactions, and other interactions not falling into any of the previous categories that we classify as hydrophobic, hydrophilic or mixed hydrophobic and hydrophilic (see Methods for a detailed definition).

For each pair of positions that covaried in both the designed and natural sequences, we computed the ten most significant transitions between amino acid pairs at those positions and classified each transition based on the mechanism of covariation. The resulting distributions of covariation mechanisms for the designed and natural pairs are shown in Figure 6B. The designed and natural covariation mechanisms distributions share similar properties, including covariation due to charge being the most common mechanism, whereas cation-pi, pi-pi and other (hydrophilic) covariation mechanisms are more rare. In both natural and designed distributions, hydrogen bonding and size covariation together account for approximately 30% of the total mechanisms. However, a number of quantitative differences exist in the distributions, including charge occurring more frequently in the designed pairs, suggesting that the design method may be over-predicting charged interactions. Additionally, in the natural pairs, size covariation is more common than hydrogen bonding covariation while the opposite is true in designed pairs. The “other” categories are also more common in the natural pairs than in the designed pairs. To better understand these differences, we split the pairs up based on the extent of their burial and compared the distributions of covariation mechanisms (Figure S4). This analysis revealed that covariation mechanism is dependent on the extent of pair burial and that buried pairs have the most significant differences between natural and designed covariation mechanisms. In natural buried pairs, the most common covariation mechanisms are size and other (hydrophobic), whereas the most common mechanisms in designed buried pairs are hydrogen bonding and size. This likely occurs due to insufficient penalization of buried polar groups during the design protocol, resulting in over-predicting polar amino acids at buried positions and therefore incorrect predictions of covariation mechanism.

To quantify how often the same covariation mechanism is used for specific pairs of positions in the designed and natural sequences, we calculated the percent of pairs sharing the same classification type in both the natural and designed sequences (percent overlap) for each type of covariation mechanism (Figure 6C). Covariation due to charge has the highest percent overlap between the designed and natural pairs, followed by hydrogen bonding, size, other (hydrophobic) and other (mixed), which have roughly equal percent overlaps. Covariation due to cation-pi and pi-pi interactions have relatively low percent overlaps between the designed and natural sequences, likely due to the fact that these types of interactions are not explicitly accounted for in the design energy function. We repeated this analysis using fixed backbone design sequences and found a decrease in the percent overlaps for size and other (hydrophobic) interactions, indicating that backbone flexibility may aid in modeling these types of covariation mechanisms (Figure S5). Taken together, this analysis provides insights into the mechanisms underlying amino acid covariation in naturally occurring proteins. Overall, the analysis shows considerable agreement between naturally occurring and designed covariation mechanisms. In some cases, it exposes pathologies in the design methods (such as the over-representation of polar amino acids in cores under-representation of cation-pi and pi-pi interactions) that can be addressed in future work using naturally occurring covariation as a reference point.

### Covarying pairs not modeled by design are more distant in three-dimensional structure and differ in amino acid pair propensities

**Figure 7:**
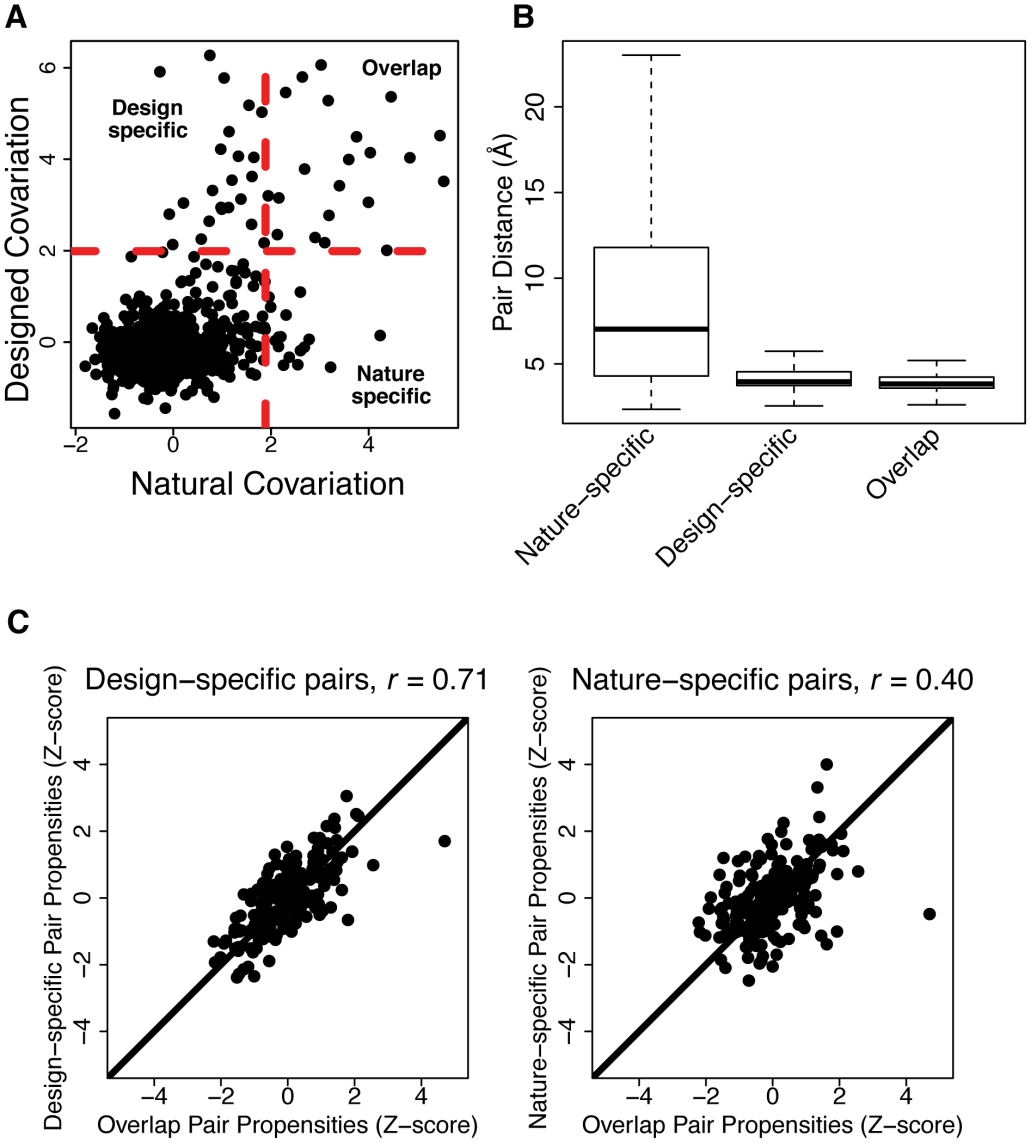
Distinguishing features of natural and designed covarying pairs. A) Example comparison of natural and designed covariation for an individual protein domain (SH3 domain). Each dot represents an amino acid pair. Dashed red lines indicate the thresholds used to identify pairs as highly covarying (two standard deviations above the mean). The indicated quadrants contain the design-specific, overlap and nature-specific pairs, respectively. B) Box plot of distances between amino acid pairs in the nature-specific, design-specific and overlap sets. Pair distances are measured as the minimum distance between heavy-atoms of two amino acids in the representative crystal structure of the domain. C) Correlation of amino acid pair propensity Z-scores between different sets of covarying pairs. The left plot shows the correlation between design-specific and overlap pairs and the right plot shows the correlation between nature-specific and overlap pairs. A Pearson correlation coefficient (r) is shown for each plot.

While computational protein design can model a significant fraction of naturally occurring covarying amino acid pairs, there remain pairs of amino acids that are highly covarying in the natural sequences but not in the designed sequences (nature-specific pairs). Moreover, there also exist pairs that highly covary in designed sequences but not in natural sequences (design-specific pairs). Figure 7A shows the classification of nature-specific, design-specific and overlap pairs for the SH3 domain. To understand the basis for these differences, we first compared these sets of pairs based on their distances in three-dimensional structure (Figure 7B). We found the design-specific and overlap covarying pairs to be significantly closer in structure than the nature-specific pairs. These results are consistent with the all-atom energy function used for generating the design sequences, which is most sensitive at short distances. The long distances in the nature-specific pairs could result from a number of factors, including interactions that bridge monomers in an oligomeric complex [37], interactions that exist in alternative conformations [37], long-range correlations in protein dynamics or from phylogenetic bias in the natural sequences. Another possibility is that in naturally occurring proteins, destabilizing substitutions (that occur in functional sites) co-vary with compensating stabilizing mutations in the protein that could be far away from the functional site.

In addition to analyzing design-specific and nature-specific pairs with respect to pair distance, we compared them based on extent of amino acid burial, the presence in interfaces or active sites, and amino acid pair propensity. We observed a slight decrease in the percent of exposed pairs in the designed-specific pairs relative to the nature-specific pairs (Figure S6), which may be due to the difficulty of accurately modeling solvent exposed interactions in protein design. We observed no difference in the design-specific and nature-specific pairs with respect to their presence in interfaces or active sites (Figure S7), suggesting that the constraints imposed by known functional sites are not responsible for the inability to model the nature-specific pairs. We observed that the amino acid pair propensities of nature-specific and overlap pairs were different, while the amino acid pair propensities of design-specific pairs were highly correlated to those of the overlap pairs (Figure 7C). The latter observation indicates that the energetic interactions leading to design-specific and overlap pairs may be similar to each other. A simple explanation may be that the design-specific pairs are equally compatible with the given protein structure, but may simply not have been sampled by nature. Such design-specific pairs may provide opportunities for engineering proteins with novel amino acid interactions, such as re-designing the specificity of protein-protein interactions.

## Discussion

Our study tested the hypothesis that the structural constraints imposed by protein architecture are a major determinant of amino acid covariation in naturally occurring proteins. If true, we reasoned that computational design methods that design sequences based on protein structure alone should be able to recapitulate amino acid covariation, provided that design predictions are sufficiently accurate. Confirming these ideas, we found a significant overlap between amino acid covariation in natural and designed protein sequences across a set of 40 diverse protein domains. These results quantify the influential role of the selective pressures for maintaining protein structure on shaping amino acid covariation. Therefore, even though correlated changes are undoubtedly important to evolve new activities and regulatory mechanisms [30], [31] the presence of covariation alone may not necessarily indicate a functional role.

Our study also illustrates how recapitulation of amino acid covariation serves as a stringent test for the ability of computational protein design methods to capture precise details of interactions between amino acids. We demonstrate that modeling backbone flexibility significantly increases the similarity between natural and designed covariation, and that this similarity is exquisitely sensitive to the mechanism used to model backbone changes. These findings indicate that protein backbone motions are required for allowing precise adjustments in amino acid interactions that enable covariation. Moreover, simulations that perform local backbone movements (Backrub and KIC) result in sequences with more natural-like covariation than simulations that perform non-local backbone movements (AbRelax, Relax, Small). Proteins may have undergone local motions similar to Backrub and KIC moves to accommodate new mutations and amino acid interactions during evolution [24], [35], [36], [41]. Such motions could have provided proteins with a mechanism to allow subtle, incremental changes to their structures without adversely affecting protein structure or protein function.

While local motions may be a common mechanism for proteins to accommodate point mutations, larger structural adjustments may be necessary for dealing with insertions or deletions. In this study, we found that a moderate degree of backbone flexibility best recapitulated natural amino acid covariation, however, the magnitude of structural variation produced by this degree of backbone flexibility was less than the structural variation among naturally occurring protein families.

This discrepancy is likely due to the assumption in the design method that the protein remains a fixed length. This is not true in naturally occurring sequences; in fact, all 40 domains in our benchmark include loop regions that have varying lengths. Mutations that change the length of a flexible loop could allow for secondary structure elements to re-orient themselves and slightly alter the tertiary structure. The accumulation of mutations in loop regions can produce significant structural diversity that cannot be modeled using a protein design method that keeps the number of amino acids in a protein constant. Future protein design methods, particularly those involving loop regions such as protein-protein interaction design or enzyme specificity design, could potentially benefit from incorporating moves that both change the conformation and length of the protein backbone.

In addition to observing significant similarity between the sets of natural and designed highly covarying amino acid pairs, we observed a high correlation in the amino acid propensities of these covarying pairs and showed that the structural mechanisms underlying covariation are similar for both natural and designed sequences. Differences between natural and designed covarying pairs highlight areas for improvement in the energy function used for protein design. For instance, cation-pi interactions, which are not explicitly accounted for in the energy function used in this study, have high propensities among naturally covarying pairs but not in designed covarying pairs. Similarly, polar amino acid pairs are more frequent in the cores of designed proteins than in naturally occurring proteins. Interestingly, we found differences in the pair propensities between nature-specific pairs and pairs that highly covary in both natural and design sequences. We also observed that nature-specific pairs tend to be more distant in three-dimensional structure. These results have implications for the field of contact prediction, as combining amino acid covariation with amino acid pair propensity information could improve the prediction of three-dimensional contacts in protein structures compared to using amino acid covariation alone. Improving methods of contact prediction would increase the accuracy of recent protein structure prediction algorithms that use amino acid covariation [29].

Unlike nature-specific pairs, design-specific pairs have amino acid propensities that are highly correlated with the amino acid propensities of pairs that covary in both natural and designed sequences. These design-specific pairs represent candidate positions for engineering amino acid interactions that have not been sampled by natural protein evolution. A practical application of this is the re-wiring of protein interaction specificity to design orthogonal protein-protein interactions for use in synthetic biology. Natural intermolecular covariation has previously been exploited to alter specificity in two component signaling systems [42]. Future work could exploit designed intermolecular covariation to re-engineer protein interactions with novel specificities that are orthogonal from naturally occurring protein-protein interactions [43] and therefore useful for synthetic applications.

## Methods

### Preparation of natural protein sequences

The protein domains used in this study were selected from the Pfam database [44] based on the following criteria: 1) at least one crystal structure of a protein containing the domain was available from the Protein Data Bank (PDB) [45], 2) at least 500 sequences of proteins from the domain were available from Pfam and 3) the domain was equal to or less than 150 amino acids in length. We selected a total of 40 domains that represented a diverse set of protein folds (Table 1). The seed alignment and the full alignment for each domain were obtained from Pfam. In order to remove highly divergent sequences with uncommon insertions or deletions, we first removed sequences from the seed alignment if they had either of the following: 1) a gap in a position where 90% of the sequences in the seed alignment did not have a gap or 2) an amino acid in a position where 90% of the sequences in the seed alignment had a gap. Next, we aligned each sequence in the full alignment to the seed alignment using MUSCLE [46] and we discarded any sequences that resulted in the creation of gaps that were not in the seed alignment. This resulted in an alignment without sequences containing uncommon insertions or deletions. Finally, we used CD-HIT [47] to filter the sequence alignments by removing sequences with 80% redundancy or greater.

### Generation of designed protein sequences

For each of the 40 protein domains, the highest resolution crystal structure of the domain was obtained from the PDB. This structure was used as a template for all the design simulations. The design method used in this study consisted of two steps: 1) the generation of a conformational ensemble and 2) the design of sequences onto each structure in the ensemble using RosettaDesign. For each of the 40 domains, 500 structures were generated for the conformational ensemble and 500 sequences were designed, one for each structure in the ensemble. Descriptions of each protocol used for generating conformational ensembles and for designing sequences are provided in Text S1 along with the corresponding Rosetta command lines.

### Amino acid covariation

Amino acid covariation was quantified using a mutual information based metric called Zpx [28]. First, the Shannon entropy is calculated at each position i as follows:
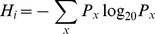
where P_x_ is the frequency of amino acid x at position i. The joint entropy is calculated between all pairs of positions as follows:
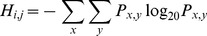
where P_x,y_ is the frequency of amino acid x and y and positions i and j, respectively. The mutual information (MI) between each pair of columns in a multiple sequence alignment, i and j, was calculated as the difference between the individual entropies and the joint entropy:
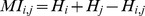
Next, the background mutual information due to random noise and shared ancestry is subtracted to obtain the product corrected mutual information (MIp) [27]:
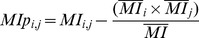
where 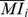 is the mean MI of position i with all other positions and 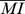 is the overall mean. This value is converted to two Z-scores, one for each column, which are multiplied together:
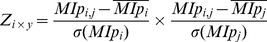
The final score, called Zpx, is the square root of the absolute value of 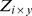. If 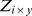 is negative, then Zpx is multiplied by −1. This normalization of MIp was demonstrated to reduce the sensitivity to misaligned regions in multiple sequence alignments, which otherwise result in artificially high mutual information scores [28]. Calculation of Zpx was implemented in Python. Direct coupling analysis (DCA) was calculated using Matlab code provided by its authors [37].

### Covariation similarity

To compare amino acid covariation between natural and designed multiple sequence alignments, Zpx was first computed for all pairs of ungapped positions in each alignment. The mean Zpx for each alignment was calculated and residue pairs with values greater than two standard deviations above the mean Zpx were considered to be covarying residue pairs. The covariation similarity between the natural and designed covarying amino acid pairs was calculated as the percent of overlap, 2C/(A+B), where A and B are the total numbers of natural and designed covarying pairs, respectively, and C is the number of pairs that covary in both natural and designed sequences. The same approach was used to calculate covariation similarity using DCA.

### Sequence recovery, entropy and profile similarity

Sequence recovery was calculated as the mean percent identity of the designed sequences to the sequence of the crystal structure used as input for the design protocol. Sequence entropy was calculated for each position as 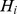 defined above. Sequence profile similarity was calculated as the mean prof_sim score [48] between each position in the natural and designed alignments. Briefly, prof_sim is the product of two scores: 1) the estimated probability that two amino acid frequency distributions represent the same source distribution and 2) the *a prior* probability of the source distribution. Using this metric, positions in designed sequences receive high prof_sim scores if both 1) their amino acid distribution is similar to the amino acid distribution at the corresponding position in the natural alignment and 2) their amino acid distribution is different than the background amino acid distribution. Calculation of sequence recovery, entropy and profile similarity was implemented in Python.

### Structural variation

Structural variation was calculated as the mean pair-wise RMSD between 10 randomly selected structures in each conformational ensemble. Natural structural variation was computed for all domains with at least 10 crystal structures in the PDB. The following 20 domains were used to compute natural structural variation: PF00013, PF00018, PF00041, PF00072, PF00076, PF00085, PF00111, PF00168, PF00169, PF00179, PF00254, PF00355, PF00439, PF00550, PF00581, PF00582, PF00595, PF01833, PF07679, PF07686. Structural alignments and RMSD calculations were performed using PyMol [49].

### Amino acid pair propensities

Amino acid pair propensities (PP) were calculated as the ratio between observed pair frequencies and the expected individual amino acid frequencies:
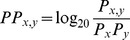
To compare amino acid pair propensities between two sets of covarying pairs, we computed the Z-score for each pair amino acid pair x,y. The Pearson correlation coefficient *r* between the two sets of Z-scores was then calculated using R [50]. Cysteines were excluded from this analysis because they rarely appear in the designed sequences.

### Covariation mechanisms

To classify the mechanisms of covariation for a pair of positions, we first computed a correlation coefficient 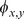 for each amino acid pair x,y [32]. We then calculated a score for all possible amino acid pair transitions (PT) between one pair x,y and another pair a,b as follows:
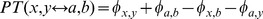
This pair transition score quantifies the significance of the transition between the amino acid pair x,y and the pair a,b. The most significant transitions are defined as those that highly favor pairs x,y and a,b but highly disfavor pairs x,b and a,y. For each pair of positions, ten pair transitions with the greatest scores were assigned one of eight classes in the following order: charge, cation-pi, pi-pi, size, hydrogen bonding, other (hydrophobic), other (hydrophilic) and other (mixed). Charge transitions involve a pair with opposite charges that either swap sign or become uncharged. A charge transition is also assigned to pair transitions that avoid like charges, for example, if x and b (or y and a) are like charges. Cation-pi transitions involve one pair with a potential cation-pi interaction but no cation-pi interaction in the other pair. Similarly, pi-pi transitions involve one pair with a potential pi-pi interaction but no pi-pi interaction in the other pair. Size transitions involve a decrease in the size of one amino acid by at least 18 Å^3^ (the volume of a methyl group) and an increase in the size of the other amino acid by at least 18 Å^3^. Hydrogen bonding transitions involve a potential hydrogen bonding interaction (hydrogen bond acceptor and donor) in one pair but not in the other pair. The three other classes are used to assign pair transitions that do not fit any of the above criteria. Other (hydrophobic) transitions are those where both pairs contain only hydrophobic amino acids, other (hydrophilic) transitions are those where both pairs contain only hydrophilic amino acids, and other (mixed) transitions are those with both hydrophobic and hydrophilic amino acids. Similarity between natural and designed was quantified using the percent overlap (defined above) for each covariation mechanism.

### Amino acid burial

Amino acid burial was defined for each position based on the number of Cβ atoms within 8 Å of the Cβ atom of the given position as follows: exposed 0–8, intermediate 9–14 and buried >14. For the covariation mechanism analysis in Figure S4, we defined pairs of positions that were buried/buried or buried/intermediate as buried pairs, exposed/buried or intermediate/intermediate as intermediate pairs, and exposed/intermediate or exposed/exposed as exposed pairs.

### Interface and active site positions

For domains with known protein–ligand or protein–protein interface information, we defined all positions with a heavy-atom within 6 Å of any heavy-atom on the binding partner as an interface position. The domains with interface information were PF00013, PF00439, PF00498, PF00691, PF00072, PF00018, PF00076, PF00249, PF00327, PF01035, PF00169, PF00550 and PF00595. For domains with known active sites, we defined all positions with a heavy-atom within 6 Å of any heavy-atom on a catalytic residue as an active site position. The domains with active site information were PF00085, PF00111, PF00355, PF00708, PF00581, and PF01451.

## Supporting Information

Figure S1 **Effect of the magnitude of structural variation on covariation similarity computed using direct coupling analysis (DCA).** Box plot showing the distributions of DCA based covariation similarity values between natural and sequences designed using backrub conformational ensembles at different temperatures.
(TIF)

Figure S2 **Effect of the magnitude of structural variation on covariation similarity for KIC and Small simulations.** Box plot showing the distributions of covariation similarity values between natural sequences and sequences designed using conformational ensembles generated with KIC moves (left) and Small moves (right) at different temperatures.
(TIF)

Figure S3 **Effects of forcing amino acid covariation on fixed backbones on different terms in the energy function.** One-body (A) and two-body (B) energy scatter plots of covarying pair energies in the context of fixed or flexible backbones for one-body (A) and two-body (B) energy terms. A description of each energy terms is provided in the Supplemental Methods.
(TIF)

Figure S4 **Effect of amino acid pair burial on covariation mechanism.** Line plots showing the percentage of each covariation mechanism for buried, intermediate and exposed pairs in natural (left) and designed (right) sequences.
(TIF)

Figure S5 **Comparison of covariation mechanisms in natural sequences and sequences designed using fixed backbone protein design.** Bar plot showing the percent overlap between natural and designed pairs for each covariation mechanism.
(TIF)

Figure S6 **Extent of amino acid burial of natural and designed covarying pairs.** Stacked bar plot showing the percent of buried, intermediate and exposed pairs in nature-specific pairs, designed-specific pairs, overlap pairs and all pairs.
(TIF)

Figure S7 **Comparison of the percent of positions in interfaces and active sites between natural and designed covarying pairs.** Scatter plot of the percent of positions in interfaces (left) and active sites (right) for natural and designed covarying pairs. A bold line is shown to denote x = y.
(TIF)

Table S1 **Covariation similarity between designed and natural sequences for each the 40 domains tested using fixed backbone protein design.** Covariation was quantified for all pairs of positions in the designed and natural sequences for each domain. Pairs were considered to be highly covarying if their covariation scores were two standard deviations above the mean or greater. Overlap pairs are those that were highly covarying in both the designed and natural sequences. The percent overlap is the fraction of overlap pairs in the combined set of highly covarying design and natural pairs. P-values were calculated using a hypergeometric distribution.
(DOCX)

Table S2 **Comparison of covariation similarity distributions for different temperature backrub simulations.** The p-values in this table were calculated using a two-tailed Student's t-test. P-values less than 0.01 are shown in bold.
(DOCX)

Table S3 **Comparison of covariation similarity distributions for different methods of generating backbone flexibility.** The p-values in this table were calculated using a two-tailed Student's t-test. P-values less than 0.01 are shown in bold. The Backrub, KIC and Small simulations shown here were run with kT values of 0.9, 1.2, and 1.2, respectively (which represents the optimal temperature for covariation similarity in each case).
(DOCX)

Table S4 **Comparison of all protein design methods used in this study based on covariation similarity, sequence profile similarity, sequence recovery, sequence entropy, structural variation and pair propensity correlation.**
(DOCX)

Text S1 **Supplementary Methods.** A description of the computational protein design protocols used in this study with corresponding command lines and a description of the energy function terms referenced in the paper.
(DOCX)
