# Supplementary figures and images for: Computational Protein Design Quantifies Structural Constraints on Amino Acid Covariation
Source: PLoS Comput Biol. 2013 Nov 14;9(11):e1003313. doi: 10.1371/journal.pcbi.1003313 (PMC3828131; doi:10.1371/journal.pcbi.1003313)

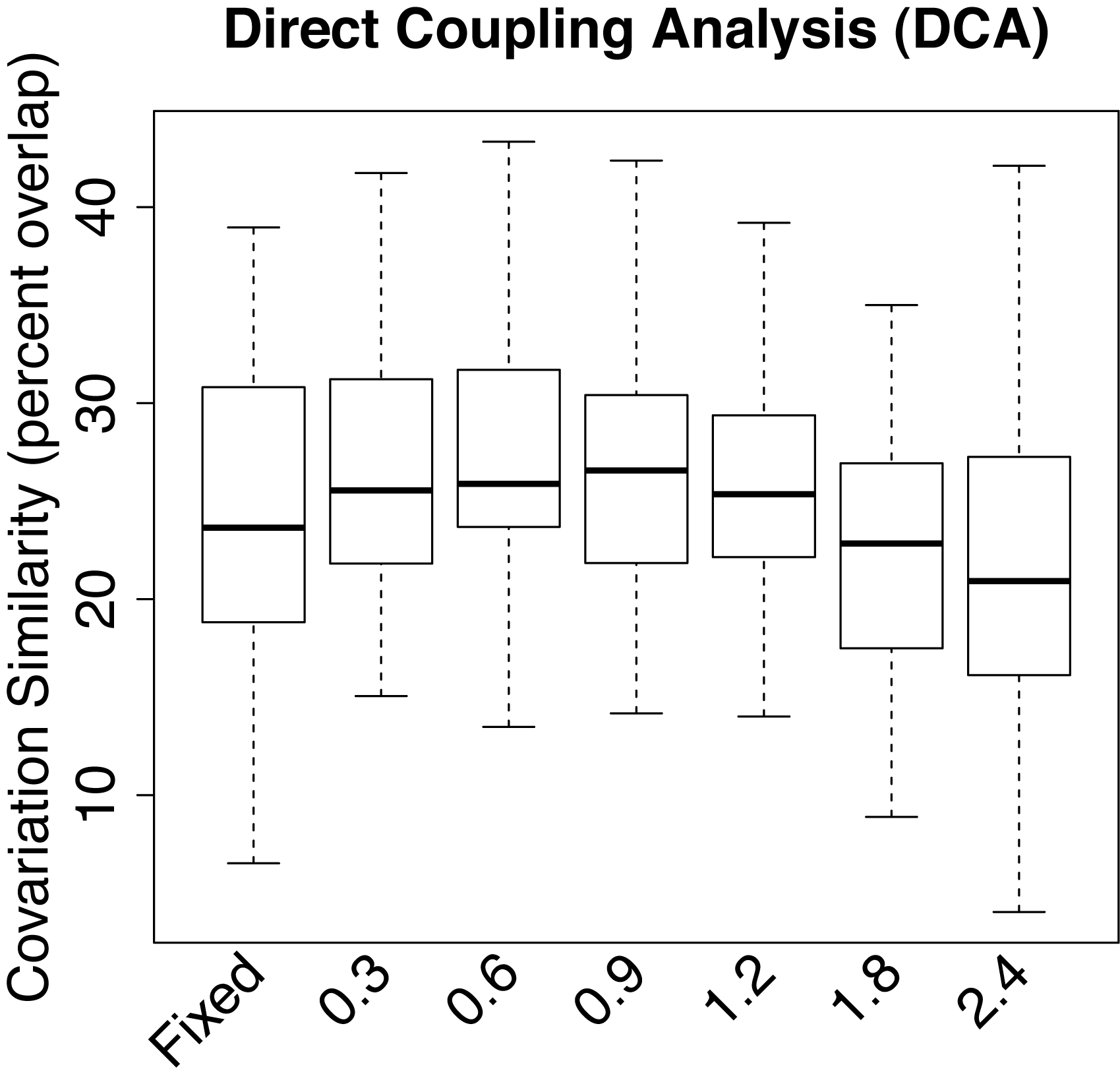

Supplement: Figure S1 — Effect of the magnitude of structural variation on covariation similarity computed using direct coupling analysis (DCA). Box plot showing the distributions of DCA based covariation similarity values between natural and sequences designed using backrub conformational ensembles at different temperatures. (TIF) [file pcbi.1003313.s001.tif]

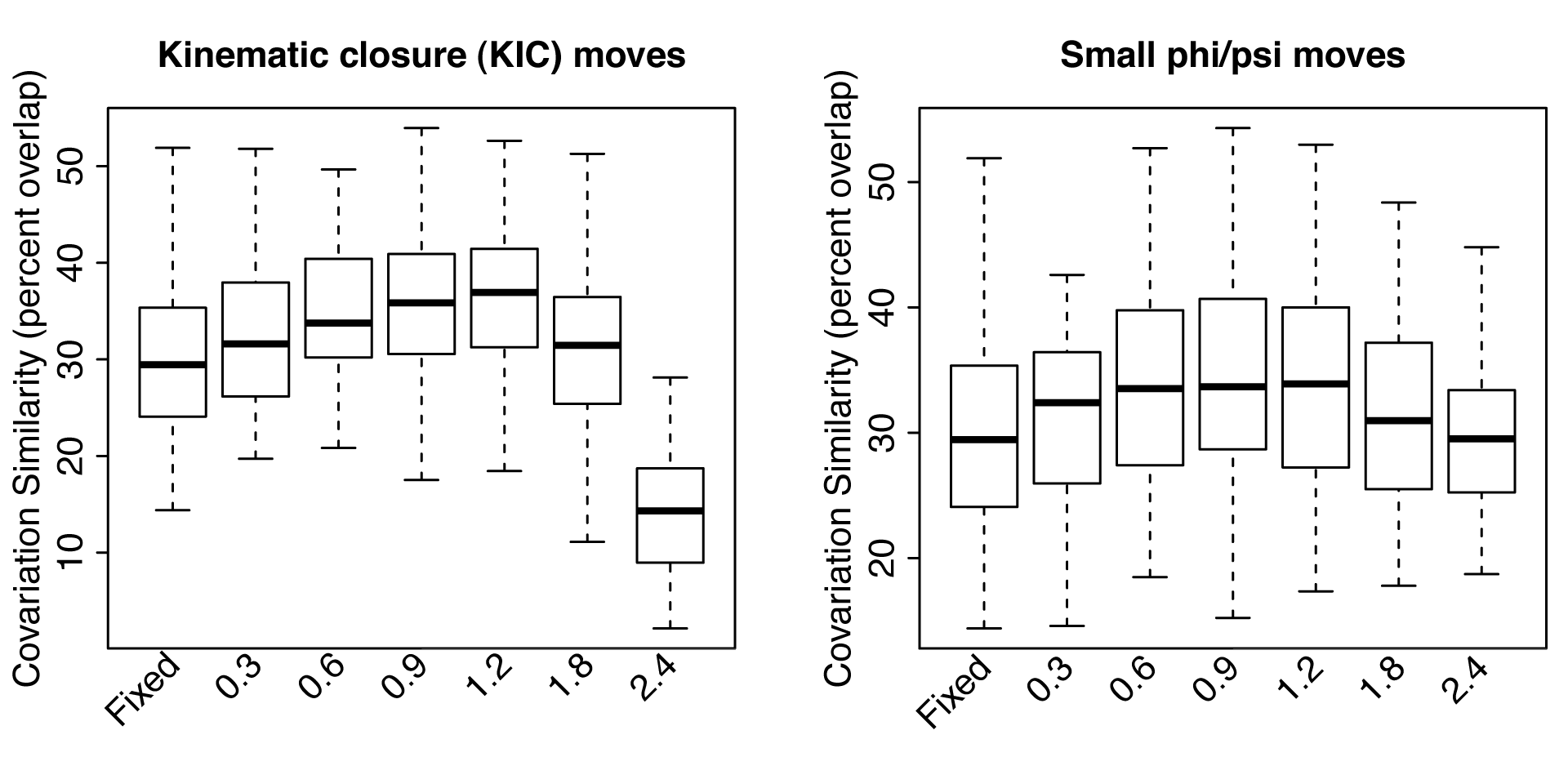

Supplement: Figure S2 — Effect of the magnitude of structural variation on covariation similarity for KIC and Small simulations. Box plot showing the distributions of covariation similarity values between natural sequences and sequences designed using conformational ensembles generated with KIC moves (left) and Small moves (right) at different temperatures. (TIF) [file pcbi.1003313.s002.tif]

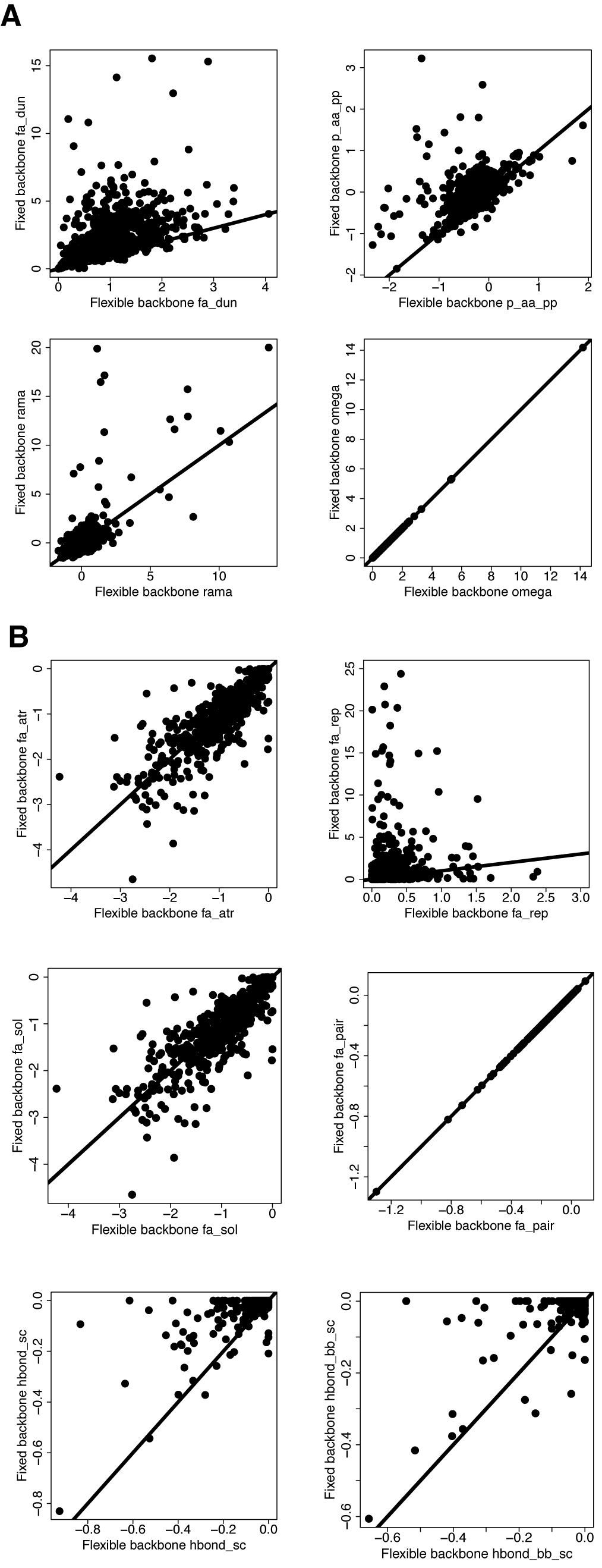

Supplement: Figure S3 — Effects of forcing amino acid covariation on fixed backbones on different terms in the energy function. One-body (A) and two-body (B) energy scatter plots of covarying pair energies in the context of fixed or flexible backbones for one-body (A) and two-body (B) energy terms. A description of each energy terms is provided in the Supplemental Methods. (TIF) [file pcbi.1003313.s003.tif]

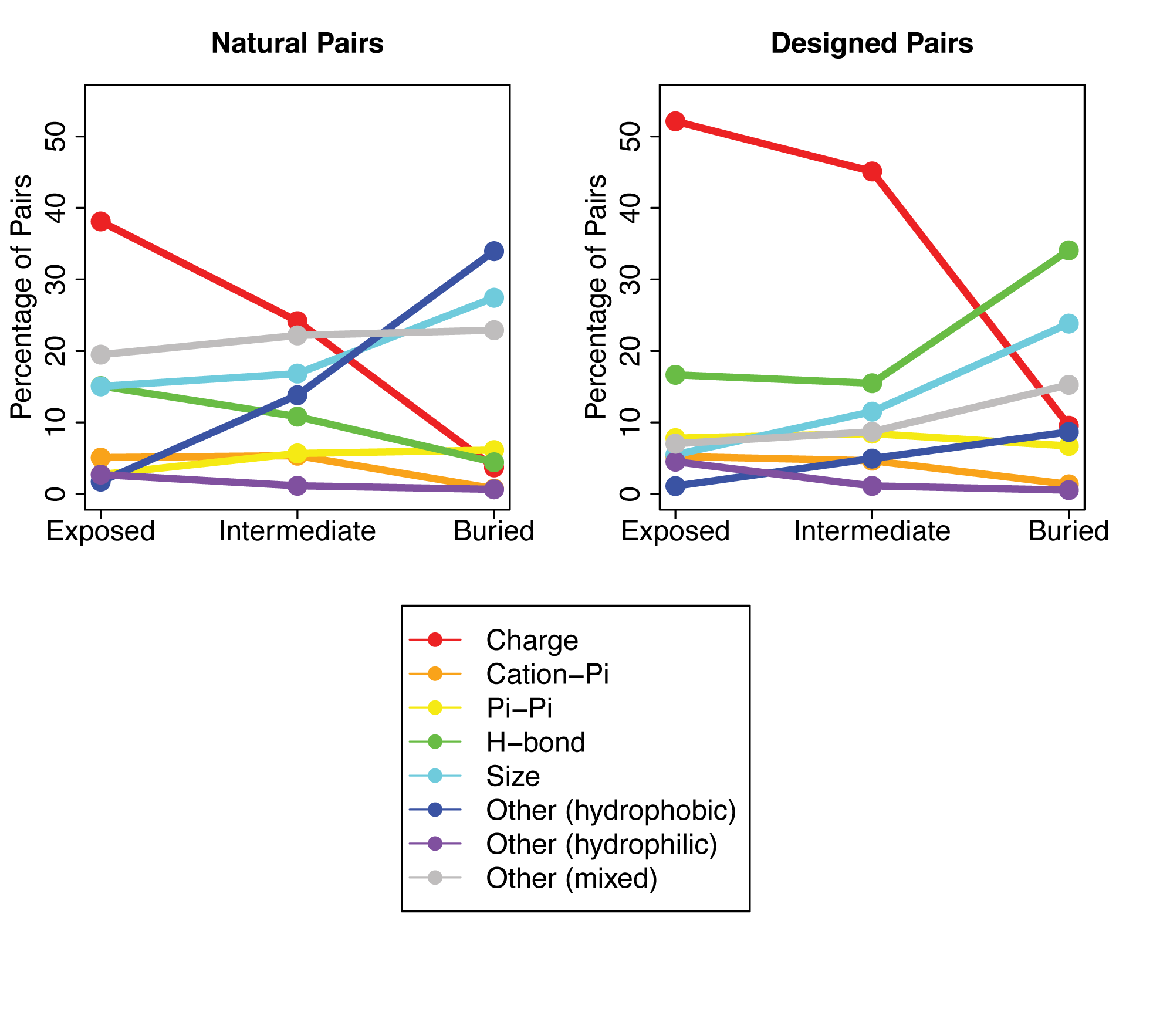

Supplement: Figure S4 — Effect of amino acid pair burial on covariation mechanism. Line plots showing the percentage of each covariation mechanism for buried, intermediate and exposed pairs in natural (left) and designed (right) sequences. (TIF) [file pcbi.1003313.s004.tif]

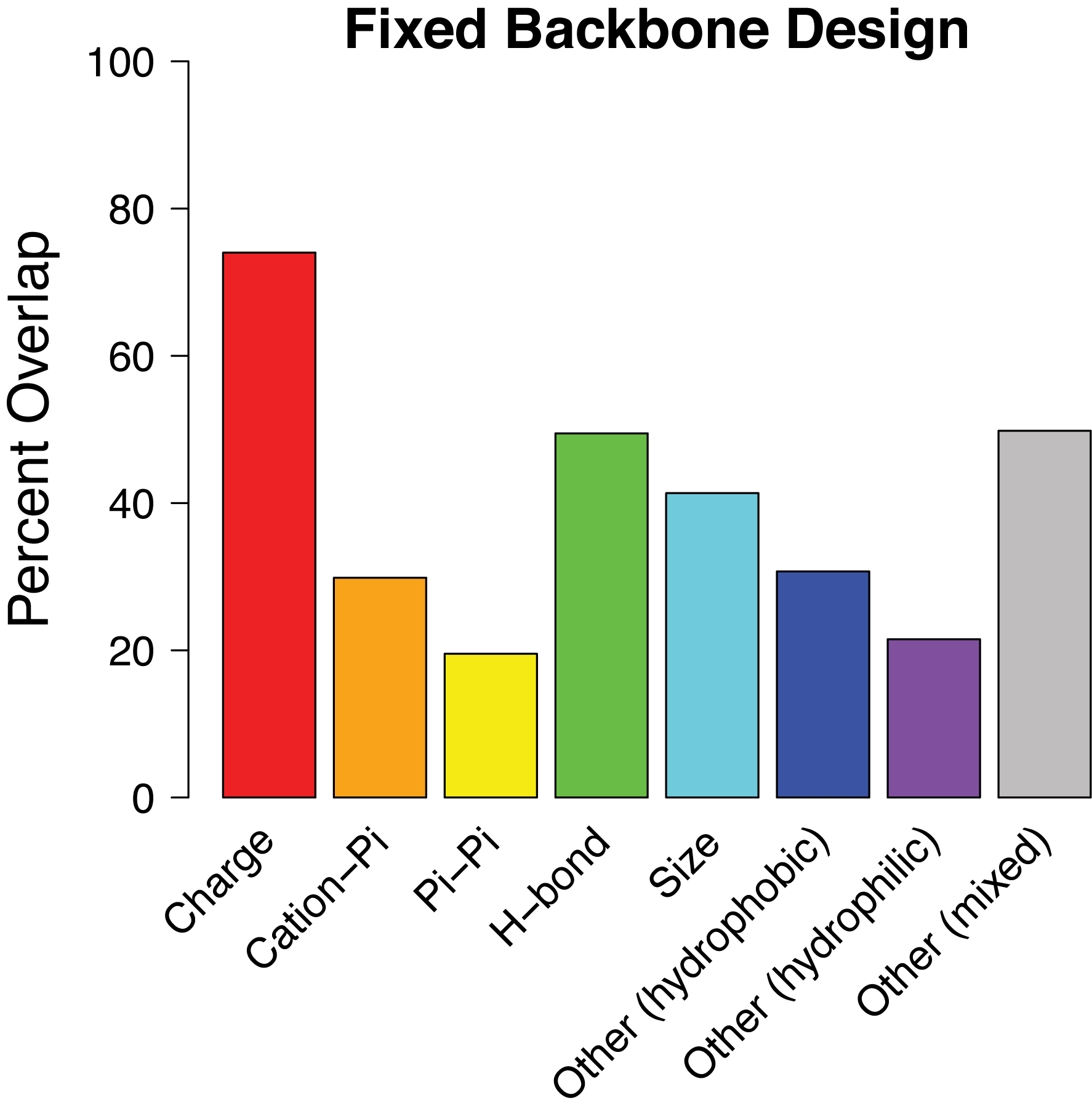

Supplement: Figure S5 — Comparison of covariation mechanisms in natural sequences and sequences designed using fixed backbone protein design. Bar plot showing the percent overlap between natural and designed pairs for each covariation mechanism. (TIF) [file pcbi.1003313.s005.tif]

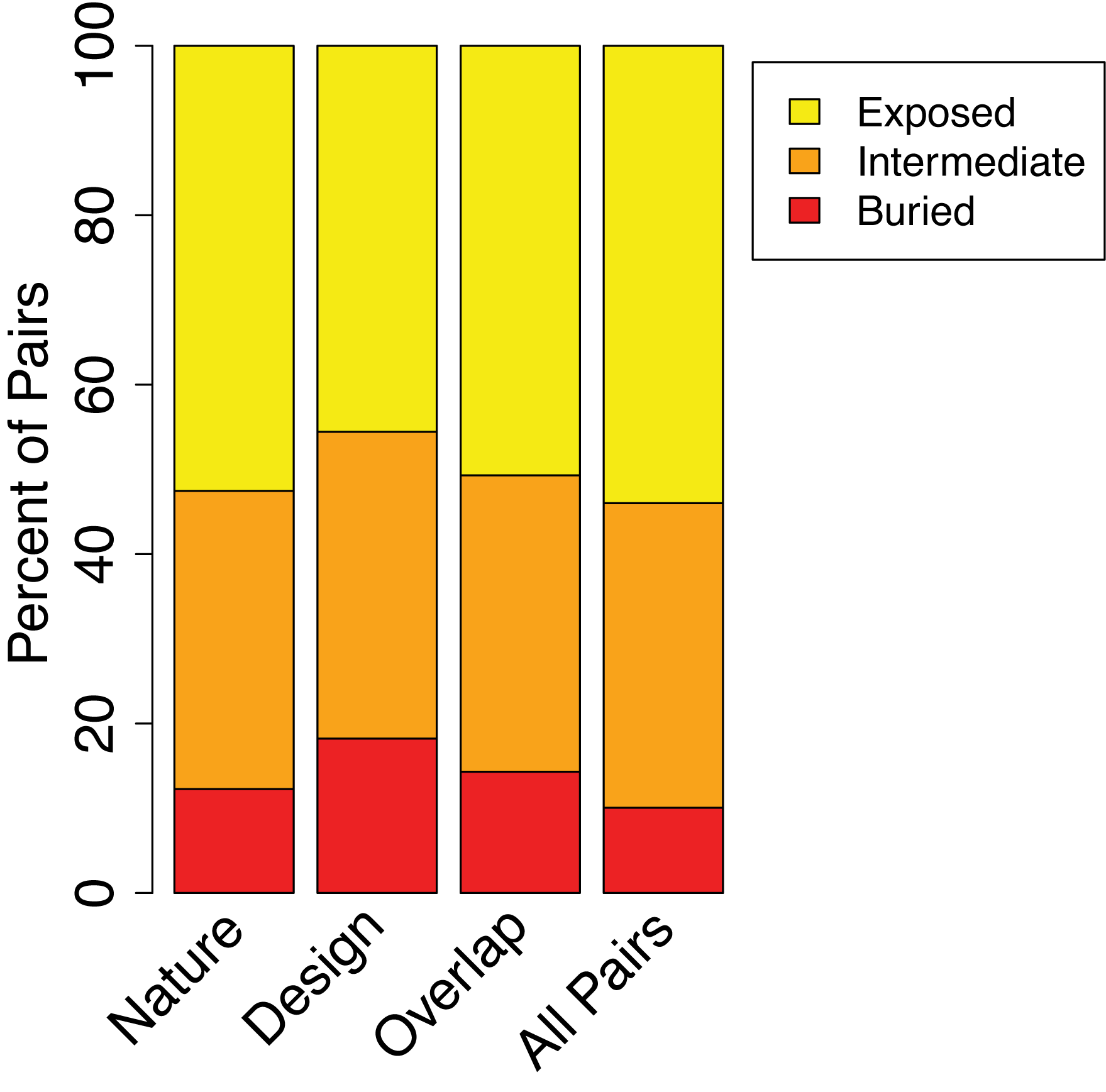

Supplement: Figure S6 — Extent of amino acid burial of natural and designed covarying pairs. Stacked bar plot showing the percent of buried, intermediate and exposed pairs in nature-specific pairs, designed-specific pairs, overlap pairs and all pairs. (TIF) [file pcbi.1003313.s006.tif]

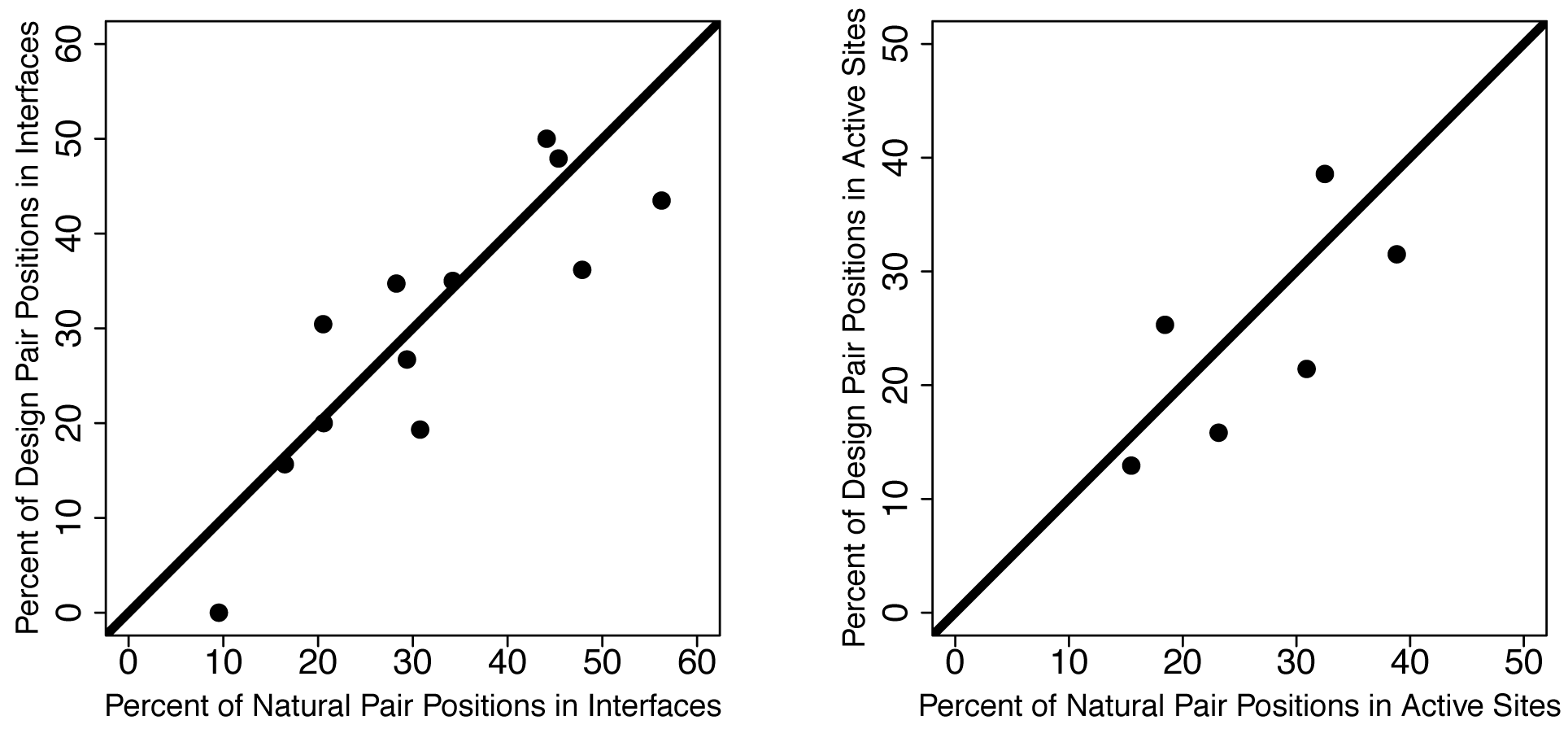

Supplement: Figure S7 — Comparison of the percent of positions in interfaces and active sites between natural and designed covarying pairs. Scatter plot of the percent of positions in interfaces (left) and active sites (right) for natural and designed covarying pairs. A bold line is shown to denote x = y. (TIF) [file pcbi.1003313.s007.tif]
