# Supplementary material for: Computational Protein Design Quantifies Structural Constraints on Amino Acid Covariation
Source: PLoS Comput Biol. 2013 Nov 14;9(11):e1003313. doi: 10.1371/journal.pcbi.1003313 (PMC3828131; doi:10.1371/journal.pcbi.1003313)
Supplement: Table S1 — Covariation similarity between designed and natural sequences for each the 40 domains tested using fixed backbone protein design. Covariation was quantified for all pairs of positions in the designed and natural sequences for each domain. Pairs were considered to be highly covarying if their covariation scores were two standard deviations above the mean or greater. Overlap pairs are those that were highly covarying in both the designed and natural sequences. The percent overlap is the fraction of overlap pairs in the combined set of highly covarying design and natural pairs. P-values were calculated using a hypergeometric distribution. (DOCX) [file pcbi.1003313.s008.docx]

| **Pfam ID** | **# Highly covarying design pairs** | **# Highly covarying natural pairs** | **# Total pairs** | **# Overlap pairs** | **Percent overlap** | **P-value** |
| --- | --- | --- | --- | --- | --- | --- |
| PF00013 | 48 | 59 | 1431 | 20 | 37 | 0 |
| PF00018 | 37 | 39 | 946 | 13 | 34 | 1.0678e-11 |
| PF00041 | 81 | 59 | 2016 | 28 | 40 | 0 |
| PF00072 | 263 | 167 | 5886 | 65 | 30 | 0 |
| PF00076 | 86 | 72 | 1653 | 41 | 52 | 0 |
| PF00085 | 154 | 104 | 4095 | 46 | 36 | 0 |
| PF00111 | 57 | 56 | 1326 | 13 | 23 | 2.1442e-08 |
| PF00168 | 98 | 79 | 2415 | 25 | 28 | 0 |
| PF00169 | 143 | 132 | 3916 | 38 | 28 | 0 |
| PF00179 | 339 | 232 | 9045 | 61 | 21 | 0 |
| PF00226 | 60 | 44 | 1275 | 19 | 37 | 1.1102e-16 |
| PF00240 | 69 | 67 | 1830 | 15 | 22 | 7.2148e-10 |
| PF00249 | 33 | 39 | 820 | 9 | 25 | 7.6141e-07 |
| PF00254 | 120 | 106 | 3403 | 35 | 31 | 0 |
| PF00313 | 78 | 59 | 1953 | 20 | 29 | 4.4409e-16 |
| PF00327 | 53 | 54 | 1326 | 20 | 37 | 0 |
| PF00355 | 158 | 85 | 3655 | 22 | 18 | 3.0109e-13 |
| PF00364 | 88 | 83 | 2211 | 28 | 33 | 0 |
| PF00381 | 150 | 116 | 3486 | 42 | 32 | 0 |
| PF00439 | 95 | 95 | 2628 | 22 | 23 | 3.8303e-14 |
| PF00486 | 104 | 74 | 2346 | 27 | 30 | 0 |
| PF00498 | 77 | 60 | 1891 | 24 | 35 | 0 |
| PF00542 | 80 | 93 | 2278 | 32 | 37 | 0 |
| PF00550 | 75 | 64 | 1596 | 10 | 14 | 1.1737e-04 |
| PF00581 | 131 | 105 | 3081 | 36 | 31 | 0 |
| PF00582 | 300 | 200 | 7140 | 65 | 26 | 0 |
| PF00595 | 105 | 83 | 2701 | 17 | 18 | 8.9649e-10 |
| PF00691 | 153 | 101 | 3240 | 48 | 38 | 0 |
| PF00708 | 146 | 112 | 3741 | 46 | 36 | 0 |
| PF01029 | 228 | 195 | 6328 | 47 | 22 | 0 |
| PF01035 | 107 | 98 | 3003 | 27 | 26 | 0 |
| PF01451 | 231 | 195 | 6328 | 44 | 21 | 0 |
| PF01627 | 89 | 93 | 2346 | 24 | 26 | 3.3307e-16 |
| PF01833 | 95 | 80 | 2556 | 25 | 29 | 0 |
| PF02823 | 112 | 87 | 2926 | 28 | 28 | 0 |
| PF04002 | 265 | 215 | 7381 | 65 | 27 | 0 |
| PF07679 | 64 | 55 | 1653 | 22 | 37 | 0 |
| PF07686 | 81 | 66 | 2080 | 13 | 18 | 9.0498e-08 |
| PF08666 | 66 | 62 | 1485 | 19 | 30 | 1.0469e-13 |
| PF12844 | 84 | 76 | 1953 | 28 | 35 | 0 |
