# Supplementary material for: Computational Protein Design Quantifies Structural Constraints on Amino Acid Covariation
Source: PLoS Comput Biol. 2013 Nov 14;9(11):e1003313. doi: 10.1371/journal.pcbi.1003313 (PMC3828131; doi:10.1371/journal.pcbi.1003313)
Supplement: Table S2 — Comparison of covariation similarity distributions for different temperature backrub simulations. The p-values in this table were calculated using a two-tailed Student's t-test. P-values less than 0.01 are shown in bold. (DOCX) [file pcbi.1003313.s009.docx]

|  | Fixed Backbone | Backrub,  kT = 0.3 | Backrub,  kT = 0.6 | Backrub,  kT = 0.9 | Backrub,  kT = 1.2 | Backrub,  kT = 1.8 | Backrub,  kT = 2.4 |
| --- | --- | --- | --- | --- | --- | --- | --- |
| Fixed Backbone | 1.00000 | 0.01313 | **0.00052** | **0.00013** | **0.00044** | 0.01936 | 0.42259 |
| Backrub, kT = 0.3 | 0.01313 | 1.00000 | 0.21757 | 0.10357 | 0.23543 | 0.85292 | 0.07856 |
| Backrub, kT = 0.6 | **0.00052** | 0.21757 | 1.00000 | 0.70490 | 0.92439 | 0.15599 | **0.00447** |
| Backrub, kT = 0.9 | **0.00013** | 0.10357 | 0.70490 | 1.00000 | 0.62537 | 0.06932 | **0.00129** |
| Backrub, kT = 1.2 | **0.00044** | 0.23543 | 0.92439 | 0.62537 | 1.00000 | 0.16738 | **0.00414** |
| Backrub, kT = 1.8 | 0.01936 | 0.85292 | 0.15599 | 0.06932 | 0.16738 | 1.00000 | 0.10947 |
| Backrub, kT = 2.4 | 0.42259 | 0.07856 | **0.00447** | **0.00129** | **0.00414** | 0.10947 | 1.00000 |
