# Supplementary material for: Computational Protein Design Quantifies Structural Constraints on Amino Acid Covariation
Source: PLoS Comput Biol. 2013 Nov 14;9(11):e1003313. doi: 10.1371/journal.pcbi.1003313 (PMC3828131; doi:10.1371/journal.pcbi.1003313)
Supplement: Table S3 — Comparison of covariation similarity distributions for different methods of generating backbone flexibility. The p-values in this table were calculated using a two-tailed Student's t-test. P-values less than 0.01 are shown in bold. The Backrub, KIC and Small simulations shown here were run with kT values of 0.9, 1.2, and 1.2, respectively (which represents the optimal temperature for covariation similarity in each case). (DOCX) [file pcbi.1003313.s010.docx]

|  | Soft | AbRelax | Fixed | Relax | Small | KIC | Backrub |
| --- | --- | --- | --- | --- | --- | --- | --- |
| Soft | 1.00000 | **0.00314** | **0.00089** | **0.00035** | **0.00000** | **0.00000** | **0.00000** |
| AbRelax | **0.00314** | 1.00000 | 0.95786 | 0.85374 | 0.02359 | **0.00138** | **0.00075** |
| Fixed | **0.00089** | 0.95786 | 1.00000 | 0.78738 | **0.00950** | **0.00028** | **0.00013** |
| Relax | **0.00035** | 0.85374 | 0.78738 | 1.00000 | 0.01875 | **0.00067** | **0.00032** |
| Small | **0.00000** | 0.02359 | **0.00950** | 0.01875 | 1.00000 | 0.28278 | 0.20008 |
| KIC | **0.00000** | **0.00138** | **0.00028** | **0.00067** | 0.28278 | 1.00000 | 0.83160 |
| Backrub | **0.00000** | **0.00075** | **0.00013** | **0.00032** | 0.20008 | 0.83160 | 1.00000 |
