# Supplementary material for: Computational Protein Design Quantifies Structural Constraints on Amino Acid Covariation
Source: PLoS Comput Biol. 2013 Nov 14;9(11):e1003313. doi: 10.1371/journal.pcbi.1003313 (PMC3828131; doi:10.1371/journal.pcbi.1003313)
Supplement: Table S4 — Comparison of all protein design methods used in this study based on covariation similarity, sequence profile similarity, sequence recovery, sequence entropy, structural variation and pair propensity correlation. (DOCX) [file pcbi.1003313.s011.docx]

| **Design Method** | **Covariation Similarity (median percent overlap)** | **Sequence Profile Similarity (median prof_sim)** | **Native Sequence Recovery (median percent identity)** | **Sequence Entropy (median Shannon entropy H)** | **Structural Variation (median backbone RMSD)** | **Pair Propensity Correlation (Pearson correlation coefficient)** |
| --- | --- | --- | --- | --- | --- | --- |
| **Native Sequences** | 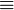100 | n/a | n/a | 0.6762 | 1.3889 | 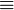1 |
| **Fixed** | 29.44 | 0.3161 | 33.11 | 0.2269 | 0.0000 | 0.3972 |
| **Backrub, kT = 0.3** | 33.97 | 0.3981 | 34.61 | 0.4755 | 0.4075 | 0.5403 |
| **Backrub, kT = 0.6** | 37.59 | 0.4171 | 29.92 | 0.5854 | 0.6070 | 0.6125 |
| **Backrub, kT = 0.9** | 37.50 | 0.4355 | 26.56 | 0.6660 | 0.8538 | 0.6740 |
| **Backrub, kT = 1.2** | 36.54 | 0.4215 | 22.89 | 0.7213 | 1.1075 | 0.5809 |
| **Backrub, kT = 1.8** | 33.62 | 0.4043 | 18.38 | 0.7819 | 1.5801 | 0.5869 |
| **Backrub, kT = 2.4** | 31.64 | 0.3887 | 16.34 | 0.8061 | 1.9430 | 0.5329 |
| **KIC, kT = 0.3** | 31.60 | 0.3802 | 38.65 | 0.3748 | 0.3305 | 0.5452 |
| **KIC, kT = 0.6** | 33.76 | 0.4033 | 36.76 | 0.4878 | 0.4781 | 0.6540 |
| **KIC, kT = 0.9** | 35.86 | 0.4190 | 34.38 | 0.5489 | 0.6059 | 0.6811 |
| **KIC, kT = 1.2** | 36.93 | 0.4291 | 32.99 | 0.6025 | 0.7184 | 0.5908 |
| **KIC, kT = 1.8** | 31.46 | 0.4283 | 27.30 | 0.7547 | 1.3621 | 0.6052 |
| **KIC, kT = 2.4** | 14.32 | 0.3937 | 20.33 | 0.8174 | 6.3083 | 0.3003 |
| **Small, kT = 0.3** | 32.40 | 0.3161 | 33.07 | 0.3310 | 0.2214 | 0.4274 |
| **Small, kT = 0.6** | 33.53 | 0.3550 | 31.05 | 0.4017 | 0.3707 | 0.5041 |
| **Small, kT = 0.9** | 33.67 | 0.3754 | 27.36 | 0.4926 | 0.4998 | 0.5510 |
| **Small, kT = 1.2** | 33.90 | 0.3788 | 25.13 | 0.5613 | 0.6592 | 0.5718 |
| **Small, kT = 1.8** | 30.97 | 0.3687 | 18.62 | 0.6415 | 2.8497 | 0.4686 |
| **Small, kT = 2.4** | 29.52 | 0.3618 | 16.52 | 0.6596 | 5.3701 | 0.4894 |
| **Relax** | 31.20 | 0.3717 | 44.96 | 0.2781 | 0.2833 | 0.4950 |
| **AbRelax** | 28.45 | 0.4454 | 34.75 | 0.6933 | 4.8012 | 0.6421 |
| **Soft** | 22.95 | 0.2986 | 33.36 | 0.1263 | 0.0000 | 0.2351 |
