# Supplementary material for: Computational Protein Design Quantifies Structural Constraints on Amino Acid Covariation
Source: PLoS Comput Biol. 2013 Nov 14;9(11):e1003313. doi: 10.1371/journal.pcbi.1003313 (PMC3828131; doi:10.1371/journal.pcbi.1003313)
Supplement: Text S1 — Supplementary Methods. A description of the computational protein design protocols used in this study with corresponding command lines and a description of the energy function terms referenced in the paper. (DOCX) [file pcbi.1003313.s012.docx]

**Text S1. Supplementary Methods.**

**Design Protocols**

**Fixed**

Fixed backbone design using RosettaDesign (Rosetta revision 39284). This protocol performs Monte Carlo simulated annealing to optimize side-chain rotamers at all positions. Rotamers of all amino acids types are allowed at any position. The following command line options were used:

./fixbb.linuxgccrelease –database rosetta_database –s input.pdb –resfile ALLAA.res –ex1 –ex2 –extrachi_cutoff 0 –nstruct 500 –linmem_ig 10 –no_his_his_pairE –minimize_sidechains

**Soft**

Fixed backbone design using RosettaDesign (Rosetta revision 39284) with softened repulsive Lennard-Jones potential. Performs the same simulation as fixbb but with a different score function weights file. The following command line options were used:

./fixbb.linuxgccrelease –database rosetta_database –s input.pdb –resfile ALLAA.res –ex1 –ex2 –extrachi_cutoff 0 –nstruct 500 –linmem_ig 10 –no_his_his_pairE –minimize_sidechains –score:weights soft_rep_design.wts

**Backrub**

Flexible backbone design that uses backrub moves to generate a conformational ensemble (Rosetta revision 39284). This protocol outputs two sets of structures; “low” structures have the lowest energy confirmation for each simulation and “last” structures have the last confirmation that was sampled in the simulation. Fixbb is used on each “last” structure to identify a low energy sequence. The value of KT was varied to generate structures with varying degrees of structural variation. The following command line options were used:

./backrub.linuxgccrelease –database rosetta_database –s input.pdb –resfile NATAA.res –ex1 –ex2 –extrachi_cutoff 0 –backrub:mc_kt KT –backrub:ntrials 10000 –nstruct 500 –backrub:initial_pack

**KIC**

Flexible backbone design that uses kinematic closure (KIC) moves to generate a conformational ensemble (Rosetta revision 40935). Fixbb is then used to identify a low energy sequence for each structure in the ensemble. The value of KT was varied to generate structures with varying degrees of structural variation. The following command line options were used:

./loopmodel.linuxgccrelease -database rosetta_database -loops:refine refine_kic -loops:input_pdb input.pdb -loops:loop_file input.loop -in:file:native input.pdb -loops:kic_max_seglen 12 -loops:outer_cycles 1 -loops:refine_init_temp KT -loops:refine_final_temp KT -loops:optimize_only_kic_region_sidechains_after_move -ex1 -ex2 -extrachi_cutoff 0 -nstruct 500 -loops:kic_recover_last -loops:max_inner_cycles 250 -loops:repack_period 250 -loops:outer_cycles 20

**Small**

Flexible backbone design that uses small phi/psi moves to generate a conformational ensemble (Rosetta revision 39284). The backrub application is used for this, but with the probability of making small moves set to 1. This protocol outputs two sets of structures; “low” structures have the lowest energy confirmation for each simulation and “last” structures have the last confirmation that was sampled in the simulation. Fixbb is used on each “last” structure to identify a low energy sequence. The value of KT was varied to generate structures with varying degrees of structural variation. The following command line options were used:

./backrub.linuxgccrelease -database rosetta_database -s input.pdb -resfile NATAA.res -ex1 -ex2 -extrachi_cutoff 0 -backrub:mc_kt KT -backrub:ntrials 10000 -nstruct 500 -backrub:initial_pack -backrub:sm_prob 1.0

**Relax**

Flexible backbone design that uses all-atom energy minimization (Relax protocol, Rosetta revision 40935) to generate a conformational ensemble. Fixbb is then used to identify a low energy sequence for each structure in the ensemble. The following command line options were used:

./relax.linuxgccrelease -database rosetta_database -s input.pdb -ex1 -ex2 -extrachi_cutoff 0 -nstruct 500

**AbRelax**

Flexible backbone design that uses fragment-based protein folding and all-atom energy minimization (AbInitioRelax protocol, Rosetta revision 42985) to generate a conformational ensemble. Fixbb is then used to identify a low energy sequence for each structure in the ensemble. This protocol was run 500 times to generate 500 structures. The following command line options were used:

./AbinitioRelax.linuxgccrelease -database rosetta_database -in:file:fasta input.fasta -in:file:frag3 aa03_05.200_v1_3 -in:file:frag9 aa09_05.200_v1_3 -abinitio:relax -relax:fast -abinitio::increase_cycles 10 -abinitio::rg_reweight 0.5 -abinitio::rsd_wt_helix 0.5 -abinitio::rsd_wt_loop 0.5 -use_filters true -psipred_ss2 input.psipred_ss2 -nstruct 500

**Energy Function Terms**

The following are brief descriptions of the energy terms referenced in this paper:

**One-body terms**

fa_dun – internal energy of side-chain rotamers as derived from the Dunbrack backbone-dependent rotamer library (probability of the rotamer given the amino acid and the backbone phi/psi dihedral angles)

p_aa_pp – probability of an amino acid for given phi/psi dihedral angles

rama – probability of phi/psi dihedral angles for a given amino acid

omega – probability of a given omega dihedral angle

ref – reference energy for each amino acid

**Two-body terms**

fa_atr – attractive component of the Lennard-Jones potential

fa_rep – repulsive component of the Lennard-Jones potential

fa_pair – statistical potential for amino acid pair preferences

fa_sol – Lazaridis–Karplus solvation potential

hbond_sc – side-chain to side-chain orientation dependent hydrogen bonding potential

hbond_bb_sc – backbone to side-chain orientation dependent hydrogen bonding potential

**Compatibility**

All command lines are compatible with Rosetta version 3.5.
